# Phytochemical combinations of lichen *Evernia prunastri* (L.) Ach. reduce drug resistance to temozolomide but not to paclitaxel *in vitro*

**DOI:** 10.3389/fphar.2025.1633978

**Published:** 2025-09-15

**Authors:** A. Shcherbakova, L. Nguyen, A. Koptina, A. Backlund, S. Banerjee, E. Romanov, G. Ulrich-Merzenich

**Affiliations:** ^1^ Medical Clinic III, Synergy and Experimental Medicine Research Group, University Hospital Bonn (UKB), Bonn, Germany; ^2^ Institute of Forestry and Nature Management, Volga State University of Technology, Yoshkar-Ola, Russia; ^3^ Pharmacognosy, Department of Pharmaceutical Biosciences, Uppsala University, Uppsala, Sweden; ^4^ Medicinal Plants Innovation Center, Mae Fah Luang University, Chiang Rai, Thailand

**Keywords:** lichens, glioblastoma, synergy, Wnt signaling, prediction, resistance

## Abstract

**Introduction:**

Temozolomide (TMZ) and Paclitaxel (PXT), crucial anti-cancer drugs for glioblastoma (GBM) and primary breast cancer (BC), respectively, face drug resistance. Therefore, we investigated the adjuvant potential of characterized extracts of the lichens *Evernia prunastri* (L.) Ach. (Epr), *Cladonia arbuscula* (Wallr.) Flot (Car) and their metabolites, evernic acid (EA) and usnic acid (UA) alone or in combination with TMZ and PTX for their immunomodulatory and chemosensitivity increasing potential.

**Methods:**

TMZ-resistant U-87 cells, MCF7 BC-cells, and normal human skin fibroblasts (HSKF) were treated with hexane (Hex), dichloromethane (DCM), and acetonitrile (ACN) extracts of Epr (EprDCM, EprACN), Car (CarHex, CarACN), and with EA and UA to measure cell metabolic activity. Molecular mechanisms were predicted using ChemGPS-NP and validated by Western blot, RNA sequencing, quantitative RT-PCR, and Wnt inhibitory factor 1 (WIF1) protein expression. Combinatory effects were calculated by Combination Index (CI) and Zero Interaction Potency methods (ZIP).

**Results:**

Extracts and selected metabolites reduced concentration-dependent cellular metabolic activity in U-87 and MCF7 cells. EprACN and EA (U-87 cells: IC_50_ 30 μg/mL), safe to HSKF, regulated key proteins in MAP kinases pathways, supporting predictions made by ChemGPS-NP. The combination EA-TMZ showed additive effects (TMZ-reduction: 3.4 fold), reduced transcription of Wnt pathway members, and increased in U-87 cells protein releases of WiF1, the central inhibitor of Wnt-signaling. Further gene expression data (GE) suggest involvement of IL-17 receptor and BDNF.

**Discussion:**

The combination EA-TMZ interacts with the Wnt pathway regulation associated with sensitizing U-87 cells, without increasing GEs of pro-inflammatory cytokines. EA deserves further investigation as an adjuvant.

## Graphical Abstract

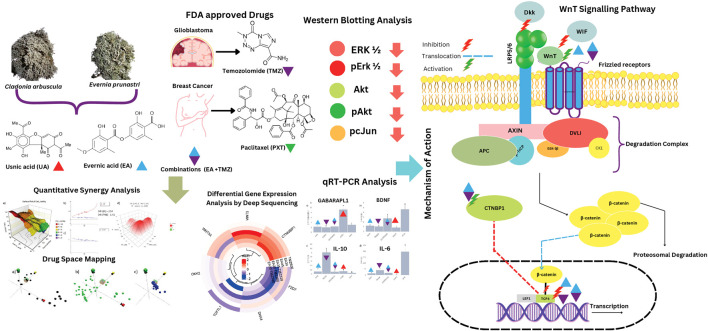

## 1 Introduction

Breast cancer (BC) is the most common cancer worldwide and ranks fifth among cancer-related deaths (Zhang et al., 2023). Projections indicate a significant increase in the global burden, with cases expected to rise by nearly 40% and deaths by 68% by 2050 if current trends continue (Liao, 2025). Brain and central nervous system cancer ranks approximately 12th in incidence and 8^th^ in mortality in Europe based on age-standardized rates (DeCordova et al., 2020). Glioblastoma (GBM), classified as a WHO Grade 4 CNS tumor according to the 2021 WHO Classification of Tumors of the Central Nervous System, represents the most aggressive form of brain cancer (Louis et al., 2021). The 5- and 10-year survival rates still remain at 5% and 2.6%, respectively (Ma et al., 2022; Alcantara Llaguno et al., 2009). The current standard of care for GBM follows the Stupp protocol, established in 2005, which includes maximal safe surgical resection, concurrent chemoradiotherapy, and adjuvant temozolomide (TMZ) (Jezierzański et al., 2024).

The poor treatment prognosis, notably GBM, has been linked to its diverse molecular profiles, resulting in distinct phenotypes also associated with TMZ-resistance (DeCordova et al., 2020). Several fundamental mechanisms contribute to GBM’s treatment resistance and incurability. The blood-brain barrier represents a critical obstacle, preventing most systemic chemotherapeutic agents from reaching therapeutic concentrations in brain tissue (Zuccarini et al., 2018). Glioblastoma stem cells (GSCs) constitute another major resistance mechanism, exhibiting enhanced DNA repair capacity, resistance to apoptosis, metabolic reprogramming, and self-renewal capacity that maintains the tumor cell population (Nowak et al., 2021; Guan et al., 2020). Methylation of the O6-methylguanine-DNA methyltransferase (MGMT) promoter plays a critical role in TMZ-resistance. Tumors with an unmethylated MGMT promoter are significantly more resistant to TMZ, and prolonged treatment may induce loss of MGMT methylation, further contributing to acquired resistance (Li et al., 2021).

Recently, Weighted Gene Co-Expression Network Analysis (WGCNA) was used to capture the molecular heterogeneity of GBM patients on the molecular level. Authors constructed an immune-related prognostic model to predict patient sensitivity to checkpoint inhibitor blockade therapy (Ma et al., 2022). The high-risk group (non-survival) was associated with epithelial-mesenchymal transition (EMT), high immune cell infiltration, immune activation, a low mutation number, and high methylation, while the low-risk group had an adverse status (Ma et al., 2022).

BC is also extremely variable in morphology and at the molecular level, necessitating combinatorial therapy modalities depending on the molecular subtype, which is defined by hormone receptor (HR) status and human epidermal growth factor receptor-2 (HER2) expression (Li et al., 2017; Kashyap et al., 2022). These include HR-positive/HER2-negative (HR+/HER2-), HR+/HER2+, HR-/HER2+, and triple-negative breast cancer (TNBC), which lacks estrogen receptor, progesterone receptor, and HER2 expression (Li et al., 2017). TNBC is particularly aggressive, accounting for approximately 10%–15% of all breast cancers but contributing to a disproportionately high percentage of breast cancer-related deaths globally, estimated at around 40% (Rosińska et al., 2024). The 5-year relative survival rate for TNBC, combining all stages, is approximately 77%, with significantly lower rates for distant metastatic disease (Baranova et al., 2022).

The current standard of care in breast cancer varies by subtype and stage. Early-stage breast cancer typically involves surgical resection, adjuvant or neoadjuvant chemotherapy, radiation therapy, hormone therapy for hormone receptor-positive tumors, and targeted therapy for HER2-positive tumors (Bravo et al., 2023; Grant et al., 2024).

The Wnt pathway is implicated in both BC and GBM, significantly contributing to treatment resistance (Zhong and Virshup, 2020), even though precise regulatory mechanisms remain unclear (Kashyap et al., 2022). Crosstalk between Wnt signaling and other pathways contributes to cancer development and spread, including resistance to pathway inhibitors (Zhong and Virshup, 2020). This understanding drives the development of novel combination therapies to minimize toxicity and resistance (Zhong and Virshup, 2020). Therefore, exploring unexamined plant extracts or phytocompounds contributes to discovering safe, effective, and novel combination therapies (Ulrich-Merzenich et al., 2017).

TMZ and paclitaxel (PTX), key chemotherapeutics for GBM and BC, respectively, are under investigation for combination therapies (Tan et al., 2020). TMZ, a DNA alkylating agent, induces cell death by causing base mismatches and DNA strand breaks (Tan et al., 2020). Ongoing 583 clinical trials worldwide explore treatments for GBM (https://clinicaltrials.gov/), including immune checkpoint inhibitors paired with CNS-penetrant or potent inhibitors (Gueble et al., 2022). Despite multimodal treatments, GBM patients face a low median survival of 12.1–16.6 months (Neff et al., 2022; Minea et al., 2024; Fekete et al., 2023), emphasizing the urgency for novel treatment strategies (Tan et al., 2020).

Taxanes, like PTX, used in BC treatment, were derived from the Pacific yew’s bark (Wani et al., 1971). PTX induces cell death by stabilizing microtubules, causing G2/M arrest and initiating apoptosis (Manthey et al., 1992). Resistance to PTX and other anti-cancer drugs (Das et al., 2021; Bukowsk et al., 2020) is common for BC, just as resistance in GBM to TMZ (Lee, 2016). The resistance of BC to PTX is thought to be a consequence of a disequilibrium in various signaling pathways, mutations in certain genes, and epigenetic deregulations (Abu et al., 2019). In particular, the genes of the ATP-binding cassette (ABC) superfamily of drug efflux, including P-glycoprotein (P-gp), are involved in the resistance to PTX by leading to an overexpression of P-gp in BC-cells (Abu et al., 2019). In MCF7 cells, aberrantly regulated expression of FOXM1 and KIF20A was associated with PTX-resistance (Abu et al., 2019; Khongkow et al., 2016). Further, the overexpression of miR-200c-3p contributed to the resistance of BC cells to PTX by an aberrant regulation of SOX2 (Abu et al., 2019).

This diversity of resistance mechanisms promotes a search for natural compounds as adjuvants (Persano et al., 2022). Therapies triggering multiple pathways (and specifically addressing crucial survival pathways) may be more promising (Sestito et al., 2018).

Lichens have been utilized in traditional medicine for ages (Crawford and Ranković, 2015). They are symbiotic organisms consisting of a fungus (mycobiont) and either algae or cyanobacteria (photobiont) (Schwendener, 2011). They are a promising source for novel organic small molecules and synergistic therapeutic strategies. *Evernia prunastri* L. and *Cladonia arbuscula* (Wallr.) Fot. were selected based on their published bioactivity. Evernic acid (EA), the main metabolite of *E. prunastri* L., has shown antimicrobial, cytotoxic, neuroprotective, and anti-inflammatory properties in prior studies (Fernández-Moriano et al., 2017; Lee et al., 2021; Gökalsın and Sesal, 2016; Shcherbakova et al., 2019). However, its role in cancer therapy remains largely unexplored. Usnic acid (UA), a prominent secondary metabolite isolated from *C. arbuscula* (Wallr.) Fot., has shown potent antiproliferative effects in several cancer types. Notably, UA exhibited promising cytotoxicity against T-47D breast cancer cells, Capan-2 pancreatic cancer cells (Einarsdottir et al., 2010), and MCF7 breast cancer cells as well (Bačkorová et al., 2011; Galanty et al., 2017; Kiliç et al., 2019; Brisdelli et al., 2013).

We investigated extracts from *E. prunastri* L. (Parmeliaceae) and *C. arbuscula* (Wallr.) Fot. (Cladoniaceae) along with their major metabolites, evernic acid (EA) and usnic acid (UA), for their potential to reduce metabolic activity in U-87 glioma and MCF7 breast cancer cells.

Both cell lines were selected as representative *in vitro* models for glioblastoma and breast cancer, respectively, due to their widespread use, well-characterized molecular profiles, and relevance to the mechanisms under investigation. U-87 cells are among the most commonly used GBM models and exhibit key features of primary glioblastoma, such as rapid proliferation, resistance to temozolomide (TMZ) (Xie et al., 2015). Similarly, MCF7 cells are commonly used for BC, characterized by estrogen receptor positivity and moderate sensitivity to chemotherapeutics like paclitaxel (PTX), making them a standard model for studying HR+ BC and mechanisms of taxane resistance (Neve et al., 2006).

U-87 and MCF7 remain widely accepted platforms for early-stage anticancer research. In this study, they were used to evaluate not only the ability of the lichen extracts and their metabolites to influence cancer cell metabolic activity, but also their potential to synergize with standard chemotherapeutics (TMZ or PTX). Extracts and combinations showing efficacy were further assessed for possible mechanisms of action. This included chemographic prediction tools, cytokine response profiling, and evaluation of their potential to modulate cellular pathways involved in drug sensitivity, forming a foundation for future translational investigations.

## 2 Materials and methods

### 2.1 Chemicals, media, and assays

Usnic acid (UA) (purity 98%), paclitaxel, resazurin tox kit, insulin (Sigma Aldrich, Germany); evernic acid (EA) (purity 98%), temozolomide (Cayman Chemical, United States); (phospho-AKT rabbit polyclonal antibody (1:2000), ß-actin rabbit polyclonal antibody (1:2000), phospho-p44/42 (Thr202/Tyr204) rabbit polyclonal antibody (1:2000), p44/42 mouse clonal antibody (1:2000), goat anti-rabbit IgG-HRP conjugated to horseradish peroxidase (1:2000) (Santa Cruz Biotechnology, United States); phospho-c-Jun (Ser73) rabbit polyclonal antibody (1:2000) (Cell Signaling Technology, United States); Minimum Essential Medium (MEM), Roswell Park Memorial Institute (RPMI) 1,640 Medium, Dulbecco’s Modified Eagle’s Medium (DMEM, fetal bovine serum (FBS), non-essential amino acids (MEM NEAA), sodium pyruvate, penicillin/streptomycin (Gibco™, United States); Human WIF-1 DuoSet ELISA (R&D Systems, United States), RNeasy MiniPlus kit (QIAGEN, Netherlands).

### 2.2 Lichens material

Samples of *E. prunastri* (L.) Ach. and *C. arbuscula* (L.) Hoffm. were collected in the Mari El Republic of Russia on the campus of the Volga State University of Technology. The lichens were identified by lichenologist G.A. Bogdanov at the Bolshaya Kokshaga Natural Reserve. The voucher specimens of the lichens were deposited at the Institute of Forestry and Nature Management, Volga State University of Technology, Yoshkar-Ola, Russia, with the references Epr_06.2012 (*E. prunastri*) and Car_06.2012 (*C. arbuscula*).

### 2.3 Extraction and characterisation

Extracts were obtained and characterized as described earlier (Shcherbakova et al., 2021). Air-dried powdered thalli of the lichens were extracted by sequential maceration with hexane, dichloromethane (DCM), or with 60% acetonitrile in water (ACN) at room temperature (RT) for 24 h with each solvent. The extracts were filtered and then concentrated under reduced pressure in a rotary evaporator Rotavapor R (Buchi Labortechnik AG, Flawil, Switzerland). The dry extracts were stored at RT until usage.

### 2.4 Chemographic prediction of the mode of action

ChemGPS-NP (http://chemgps.bmc.uu.se) provides a multidimensional (8D) map of physicochemical property space. On this map, molecules are positioned based on their estimated physico-chemical properties. Compounds with similar structures and hence properties are positioned on the map in mutual proximity. Thus positions and distances between compounds can be used to predict their biological activities. This method has been specifically validated for anti-cancer modes of action as well as for a broad range of other experimentally demonstrated activities (Buonfiglio et al., 2015). It was used to predict possible modes of action of EA.

### 2.5 Cell culture and cytotoxicity assay

#### 2.5.1 Cell lines and culture

Human primary glioblastoma (U-87), human breast adenocarcinoma (MCF7), and human skin fibroblast (HSKF) cell lines were purchased via Cell Line Services (CLS) from the German Collection of Microorganisms and Cell Cultures (DSMZ) and Promocell and grown as described earlier (Ammar and Ulrich-Merzenich, 2017). HSKF was included as a non-malignant cell line to assess the general cytotoxicity and selectivity of the tested extracts, metabolites, and compounds, distinguishing between broad cellular effects and specific anti-cancer activity.

#### 2.5.2 Metabolic activity assay as measure for cell viability

Cells (5 × 10^3^ cells/well) were seeded into 96-well plates and treated as described (Ulrich-Merzenich et al., 2017). For the treatment with the CarHex, CarACN, EprDCM, and EprACN extracts, a concentration range of 6.25–100 μg/mL was used. For EA and UA, the tested concentration range was 4.15–66.46 μg/mL and 4.30–68.86 μg/mL, respectively (corresponding to 12.5–200 µM). TMZ was tested in a range of 9.71–155.32 μg/mL (50–800 µM), and PTX in a range of 21.35–341.56 μg/mL (25–400 µM). All treatments were performed for 24 h. Metabolic activity of the cells, as an indirect measure of cell viability, was measured by resazurin fluorometric assay (Sigma) as described (Ulrich-Merzenich et al., 2017). The concentration range was selected based on the published data (Einarsdottir et al., 2010; Kiliç et al., 2019; Brisdelli et al., 2013; Emsen et al., 2018; Bézivin et al., 2004). The 24-h time point was selected for all viability assays to ensure consistency and comparability across all experimental conditions. This time frame is widely used for resazurin-based cytotoxicity assays and is sufficient to capture early drug-induced effects on cell viability, as well as to minimize secondary effects such as nutrient depletion or over-confluence in culture.

### 2.6 Western blot analysis

Western blot analysis was performed to investigate the expression and phosphorylation levels of Akt, Erk1/2, and c-Jun in U-87 cells treated with EprACN or EA as described (Ulrich-Merzenich et al., 2007) and in Supplementary Material S1. Antibody (Ab) details (pAKT, pErk1/2, Erk 1/2, p-cJun, ß-actin, secondary Abs) are provided in Supplementary Material Table S1.

### 2.7 Synergy screening

Cells (as described under Section 2.5.2) were treated with 7 different combinations of metabolites/compounds. Concentrations for the combinations were chosen based on the results with the single extracts/metabolites (Chou, 2010) (see also Section 3.1 and Supplementary Material S2). A total of five concentrations were chosen with the following concentration ranges for the different metabolites/drugs: 1) TMZ (9.75–155.32 μg/mL = 50–800 µM) and EprACN (6.25–100 μg/mL); 2) TMZ (9.75–155.32 μg/mL = 50–800 µM) and EA (4.15–66.46 μg/mL = 12.5–200 µM); 3) TMZ (9.75–155.32 μg/mL = 50–800 µM) and UA (4.30–68.86 μg/mL = 12.5–200 µM); 4) PTX (5.34–85.39 μg/mL = 6.25–100 µM) and EprACN (6.25–100 μg/mL); 5) PTX (5.34–85.39 μg/mL = 6.25–100 µM) and EA (4.15–66.46 μg/mL = 12.5–200 µM); 6) PTX (5.34–85.39 μg/mL = 6.25–100 µM) and UA (4.30–68.86 μg/mL = 12.5–200 µM); 7) TMZ (62.13–124.26 μg/mL = 320–640 µM) and EA (6.65–13.29 μg/mL = 20–40 µM). For details, see Supplementary Material S3. Drug combinations were tested in comparison to their respective controls.

#### 2.7.1 Synergy calculation

Synergism was calculated using the CompuSyn software (https://compusyn.software.informer.com/) based on the Chou and Talalay Combination Index (CI) method (Chou, 2010) and by the web application “SynergyFinder” (v.1) employing the Zero Interaction Potency (ZIP) model (Ianevski et al., 2017). For the CI and the Dose Reduction Index (DRI) calculation, the following ratios of the combined drugs were used: 1) TMZ: EP – 1.5:1 (c:c); 2) TMZ: EA – 8:1 (c:c); 3) TMZ: UA – 8:1 (c:c); 4) PTX: EP – 1:1.2 (c:c); 5) 4) PTX: EA – 1:2 (c:c); 6) 4) PTX: UA – 1:2 (c:c); 7) TMZ: EA – 16:1 (c:c).

### 2.8 RNA deep sequencing of primary glioblastoma cells (U-87)

#### 2.8.1 Cell culture

U-87 cells (106 cells/well) were seeded in 6-well plates and stimulated for 24 h with the following treatments: 1) EA (9.97 μg/mL = 30 µM); 2) EA (14.95 μg/mL = 45 µM); 3) TMZ (116.49 μg/mL = 600 µM); 4) EA (6.65 μg/mL = 20 µM) + TMZ (73.78 μg/mL = 380 µM); 5) EA (6.65 μg/mL = 20 µM) + TMZ (112.61 μg/mL = 580 µM); 6) EA (11.63 μg/mL = 35 µM) + TMZ (62.13 μg/mL = 320 µM); 7) untreated controls.

#### 2.8.2 RNA isolation and sequencing

RNA was extracted as described (Ulrich-Merzenich et al., 2017) 100 ng/μL was used for RNA sequencing. The RNA sequencing was performed by the NGSCore Facility of the University Hospital Bonn. RNASeq data were deposited into the Gene Expression Omnibus database under accession number GSE245919 (URL: 154 https://www.ncbi.nlm.nih.gov/geo/query/acc.cgi?acc=GSE245919).

#### 2.8.3 Data evaluation

Data evaluation was performed according to the guidelines provided on the Galaxy Training website (https://training.galaxyproject.org/) and with Ingenuity Pathway Analysis (QIAGEN IPA). GeneMANIA (Warde-Farley et al., 2010) was used to construct the network based on data on co-expression, genetic interaction, and pathways (https://genemania.org/).

### 2.9 Quantitative RT-PCR (qRT-PCR)

The same RNA samples analyzed by RNASeq were used for quantitative reverse transcription (qRT-PCR) as described earlier (Abdel-Aziz et al., 2015). See also Supplementary Material S3, S4.

### 2.10 Protein Wnt-inhibitory factor 1 (WIF1) release

Cells were treated as described under 2.5.2. Treatment was either with EA (6.65, 13.29, and 19.94 μg/mL = 20, 40, and 60 µM) or TMZ (58.25, 87.37, and 116.49 μg/mL = 300, 450, and 600 µM) alone or in combinations. The WIF1 release was determined by Human WIF-1 DuoSet ELISA (R&D Systems).

### 2.11 Statistical analysis

All values are expressed as mean ± SEM of three independent experiments. Experiments were performed with at least 3 replicates for each condition, if not otherwise mentioned. Statistical analyses were performed with SigmaStat (v.4.0) (http://www.systat.de/SigmaStat4_PR.html) and Origin 2018 software (https://www.originlab.com/origin) packages.

## 3 Results

### 3.1 Effects of lichen extracts and metabolites on cancer cells and fibroblasts

The ability of the lichen extracts, metabolites, and reference drugs to reduce the metabolic activity of TMZ-resistant U-87 and MCF7 cell lines and normal human skin fibroblasts (HSKF) is displayed in Table 1 and Supplementary Material S2.

**TABLE 1:** Effect of lichen extracts, lichen metabolites, and reference drugs on cell viability.

Extract/Compound	IC_50_, fibroblasts	IC_50_, U-87	SI_U-87	IC_50_, MCF7	SI_MCF7
*E. prunastri* (EprDCM)	29 ± 5 μg/mL	13 ± 3 μg/mL*	2.3 ± 0.19	72 ± 8 μg/mL*	0.4 ± 0.02
*E. prunastri* (EprACN)	79 ± 6 μg/mL	31 ± 6 μg/mL*	2.6 ± 0.15	107 ± 13 μg/mL	0.7 ± 0.03
*C. arbuscula* (CarHex)	44 ± 2 μg/mL	35 ± 3 μg/mL*	1.3 ± 0.03	85 ± 14 μg/mL*	0.5 ± 0.02
*C. arbuscula* (CarACN)	16 ± 2 μg/mL	33 ± 2 μg/mL*	0.5 ± 0.02	67 ± 7 μg/mL*	0.2 ± 0.01
Evernic acid (EA)	>66.46 μg/mL (200 μМ)	20.60 ± 2.99 μg/mL 62 ± 9 µM*	3.3 ± 0.13	>66.46 μg/mL (200 µM)	1.0
Usnic acid (UA)	>68.86 μg/mL (200 μМ)	69.90 ± 5.16 μg/mL 203 ± 15 μМ	1.0 ± 0.02	57.50 ± 9.30 μg/mL 167 ± 27 μМ	1.2 ± 0.05
Temozolomide (TMZ)	>155.32 μg/mL (800 μМ)	115.13 ± 6.40 μg/mL (593 ± 33 μМ)	1.4 ± 0.02	—	
Paclitaxel (PTX)	>341.56 μg/mL (400 μМ)	—		116.13 ± 12.81 μg/mL 136 ± 15 μМ	3.0 ± 0.1

The effect is represented as an IC_50_ value. SI, selectivity index defining the ratio between IC_50_s of fibroblasts and cancer cells, as higher the SI as better the selectivity. For experimental details see Material and Methods.

*p-value <0.05 in comparison to TMZ or PTX. Experiments were performed with 3 replicates.

The IC_50_ of TMZ against U-87 cells was high, confirming resistance to TMZ (Lee, 2016). All lichen extracts and metabolites significantly reduced the metabolic activity of U-87 cells. EprDCM had the highest potency, however, it also affected HSKF at a similar concentration, showing undesirable off-target action. EprACN, CarHex, and CarACN showed comparable reduction in U-87 cell metabolic activity. CarACN showed a high effect on HSKF, while EprACN demonstrated favorable specificity with a high selectivity index (SI) for U-87.

EA and UA did not significantly affect the metabolic activity of HSKF at concentrations up to 66.46 μg/mL and 68.86 μg/mL (equivalent to 200 µM), respectively. In U-87 cells, EA demonstrated greater activity and more favorable response comparing to UA. The results indicated that while the compounds were effective against cancer cell lines, they exhibited reduced activity toward HSKF cells, suggesting a degree of selectivity toward malignant cells.

The lichen extracts did not reduce the metabolic activity of MCF7 with the SI value below 1, indicating a lack of selectivity. Both EA and UA showed activity against MCF7 cells, but their SI was comparatively lower than that of PTX, leading to the discontinuation of further experiments with MCF7 cells. In the next step, combinations were investigated.

### 3.2 Combinatorial effects of lichen metabolites with TMZ or PTX

**FIGURE 1:**
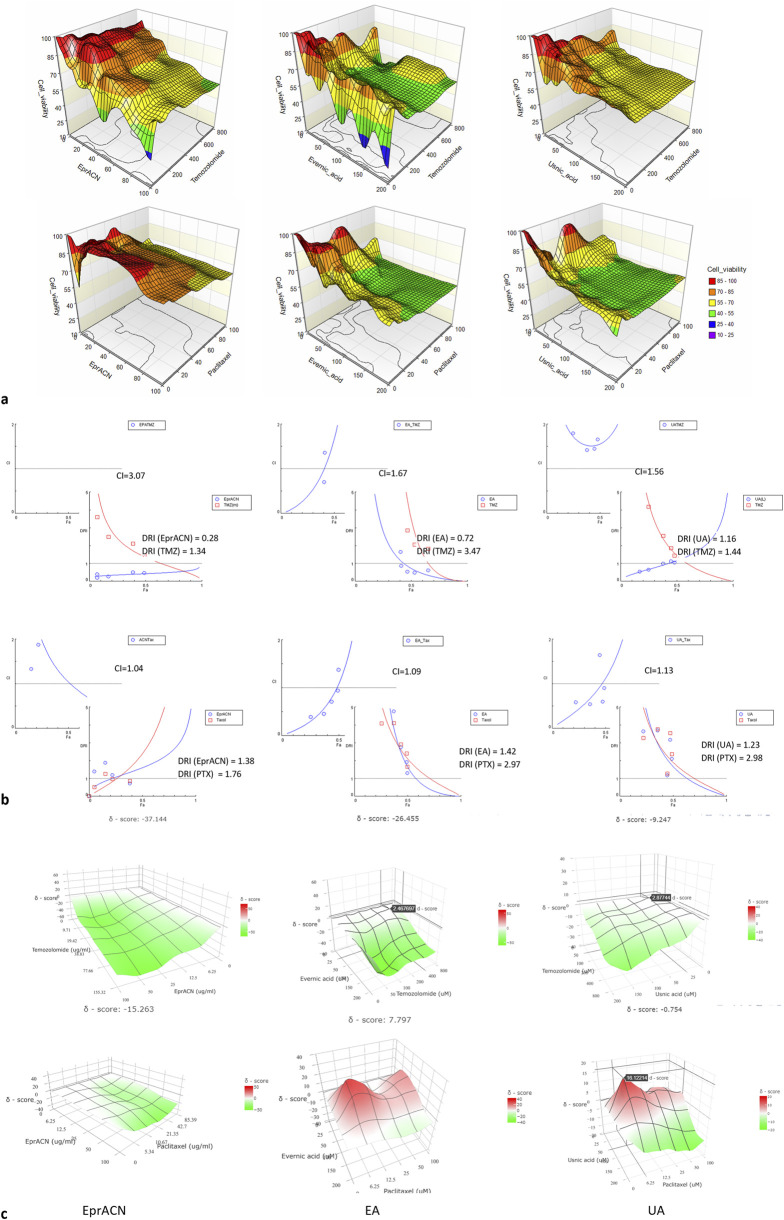
Synergy assessments **(a)** Surface plots illustrating the cell viability expressed as a percentage of the control (Z-axes) depending on the treatment with the combination of TMZ or PTX (X-axes) and EprACN or EA or UA (Y-axes) at different concentrations. **(b)** Plots indicating the relation of CI and DRI to Fa. The CI < 1 denotes synergy, CI = 1 addition, and CI > 1 antagonism. **(c)** Surface plots illustrating the δ-score (Z-axes) indicating synergy (red), additive effect (white), or antagonism (green) for the combinations of TMZ or PTX (Y-axes) with EprACN, EA, or UA (X-axes) at various concentrations. Experiments were performed with 3 replicates.

Figure 1a demonstrates the effects of combining TMZ or PTX with EprACN, EA, or UA on the metabolic activity of U-87 and MCF7 cells. The combination Index (CI) and the Dose Reduction Index (DRI) are shown in Figure 1b and the δ-score in Figure 1c.

The combination of EprACN with TMZ exhibited antagonism (CI = 3.07) and with PTX additive effects (CI = 1.04) (Figure 1b). Despite the additive effect of EprACN-PTX, metabolic activity remained above 50%. Consequently, combinations involving EprACN were not pursued. The UA-TMZ combination also displayed limited modulation of the metabolic activity (Figure 1a), and was therefore excluded from further investigations.

Although the EA-TMZ combination demonstrated antagonism, EA increased the sensitivity of U-87 cells to TMZ by 3.4 times (DRI = 3.4). Synergistic effects were observed at Fa < 0.5 (Figure 1b) with a CI of 0.97 for the combination of EA (8.31 μg/mL = 25 µM) and TMZ (77.66 μg/mL = 400 µM) at a 1:4 ratio (Figure 1c), resulting in approximately 50% reduction in metabolic activity (Figure 1a).

Effects of the EA-PTX and UA-PTX combinations were similar, with enhanced effectiveness leading to up to 60% in metabolic activity reduction (Figure 1a). These combinations had a primarily additive effect (Figure 1b, CI ≈ 1), transitioning to synergistic effects at concentrations of EA (4.15–33.23 μg/mL = 12.5–100 µM) – PTX (5.34–85.39 μg/mL = 6.25–100 µM) and UA (4.30–34.43 μg/mL = 12.5–100 µM) – PTX (5.34–85.39 μg/mL = 6.25–100 µM) (Figure 1c, δ-score > 0).

**FIGURE 2:**
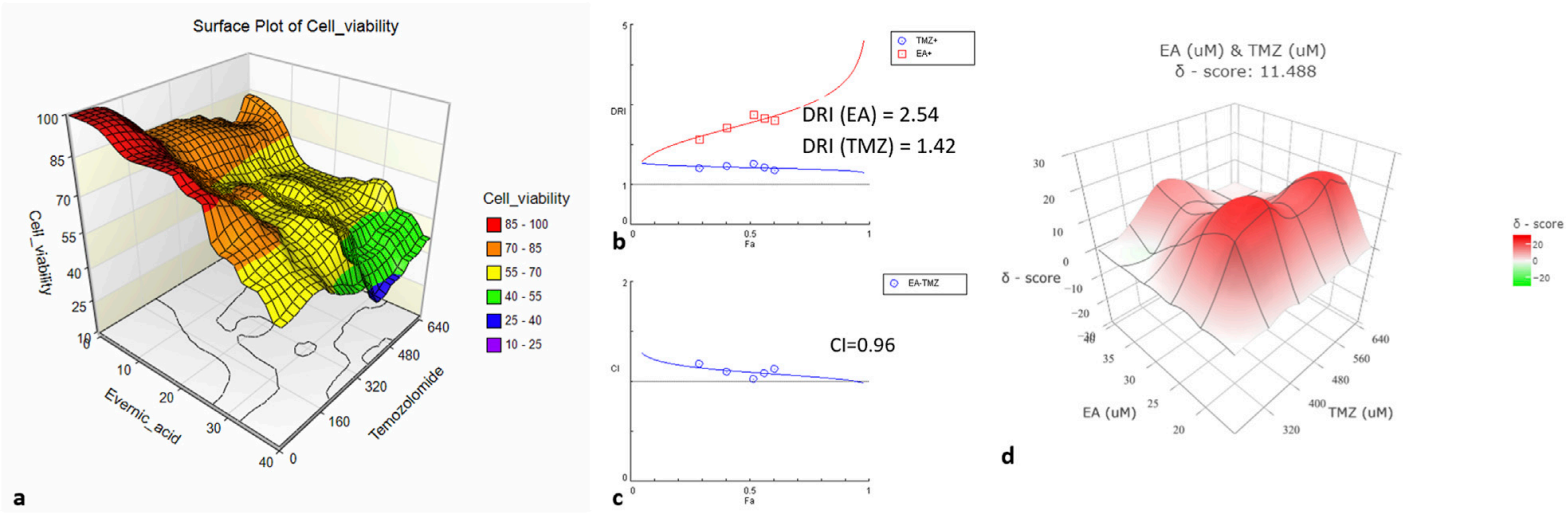
U-87 cell viability and synergy calculations for the combinations of TMZ with EA at a ratio of 1:16. **(a)** Surface plot illustrating the cell viability as a percentage of the control (Z-axes) with the combination of TMZ (X-axes) and EA (Y-axes) at different concentrations. **(b)** The plot illustrates the relation of DRI to Fa. **(c)** The plot illustrates the CI according to Fa. **(d)** The surface plot illustrates the δ-score (Z-axes) indicating synergy for TMZ (Y-axes) with EA (X-axes) combination at different concentrations. Experiments were performed with 3 replicates.

Since synergistic effects are influenced not only by drug concentrations but also by their ratio (Ammar and Ulrich-Merzenich, 2017), further research focused on the ratio (1:16) that demonstrated synergy (Figure 2).

**FIGURE 3:**
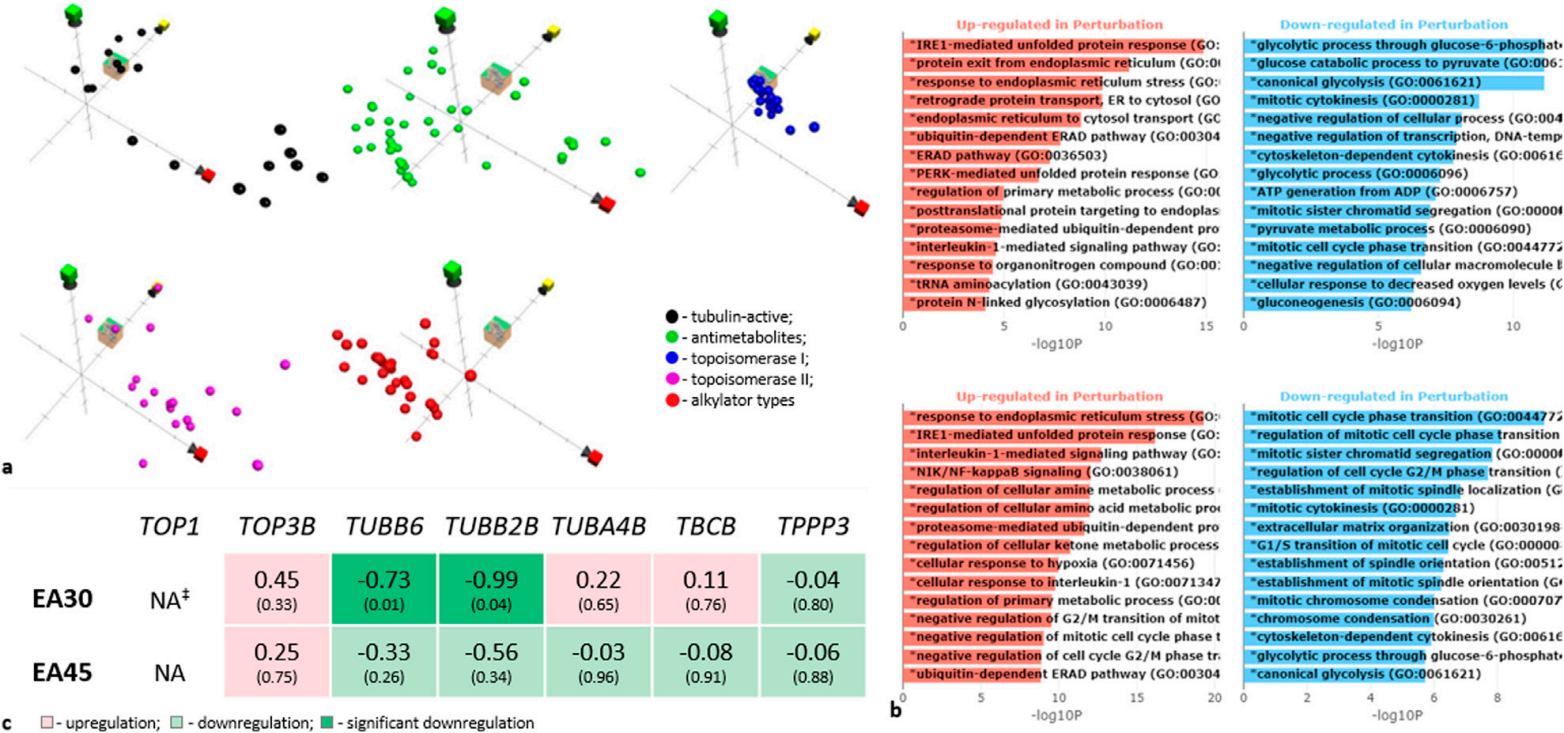
Investigation of the mechanism of action of *Evernia prunastri* ingredients. **(a)** Position in chemical property space of EA (cube), compared to reference sets of compounds in ChemGPS. The axis markers: red–size-related properties, yellow–conjugation and aromaticity-related properties, and green–lipophilicity and solubility-related properties. **(b)** Gene ontology enrichment analysis bar plot based on RNA deep sequencing data. Y-axis indicates significantly enriched GO biological processes, X-axis shows -log_10_ (p-values). Upper panel -EA 30 µM (9.97 μg/mL), lower panel -EA 45 µM (14.95 μg/ml). For details see Supplementary Material S5. **(c)** Gene sequencing data for tubulin-encoding genes. ^‡^NA: the gene is “not available” or the gene was excluded from analysis because it contained an extreme count outlier–large counts (during the gene expression analysis in Galaxy). Experiments were performed with 3 replicates.

At a ratio of 1:16, the TMZ-EA combination reached the maximum reduction of metabolic activity (75%). The IC_50_ of the drug combination was lower than the IC_50_ values of the single drugs (Figure 2a). Both metabolites showed a dose reduction (DRI > 1), and the CI value indicated additive effects (Figures 2 b,c). The δ-score demonstrated synergistic effects (Figure 2d). These promising results led to the investigation of the potential mechanisms underlying EA’s action (Figure 3).

### 3.3 Mechanistic insights into EA’s mode of action

In a first step, we used ChemGPs for a chemographic prediction of EA’s mode of action. The Euclidean distance calculation over all eight dimensions of the ChemGPS-NP chemical property space suggests that EA’s most probable mode of action is either topoisomerase I inhibition or disturbance of tubulin activity (Figure 3a). EA shares chemical properties with topoisomerase I (TOP1) inhibitors. Few TOP-1 inhibitors are semi-synthetic derivatives of the plant alkaloid camptothecin, stabilizing TOP1-DNA cleavable complexes (Top1cc) (Thomas and Pommier, 2019/06). In a second step, we compared the prediction with the RNAseq data analyses of the experiments. Here, the level of TOP1 was insignificant (Figure 3c). Thus, EA may interact with TOP1 either by blocking the DNA-helix or by disrupting, e.g., electrostatic interactions on the surface of TOP1 by binding to the active site.

Tubulin-active compounds bind to the tubulin microtubules, affecting their dynamics (Janke and Magiera, 2020). Although we did not examine the polymerization of microtubules and their dynamics, we evaluated the transcription of tubulins. EA decreased transcription of the β-tubulins *TUBB6* and *TUBB2B* (Figure 3c). Since microtubules consist of α-β tubulin heterodimers (Janke and Magiera, 2020), a downregulation of β-isotypes may change the microtubule formations and prevent cell division. The Gene Ontology analysis of genes regulated by EA demonstrated an involvement of mitotic cell cycle phase transition, mitotic sister chromatid segregation, the establishment of mitotic spindle localization, mitotic cytokinesis, and mitotic chromosome condensation in such an activity (Figure 3b). This supports the prediction for EA to be a tubulin-active compound (see also Supplementary Material S5 for results of the Expression of genes coding tubulin-related proteins and Supplementary Material S6 for results of the Gene Ontology analysis).

**FIGURE 4:**
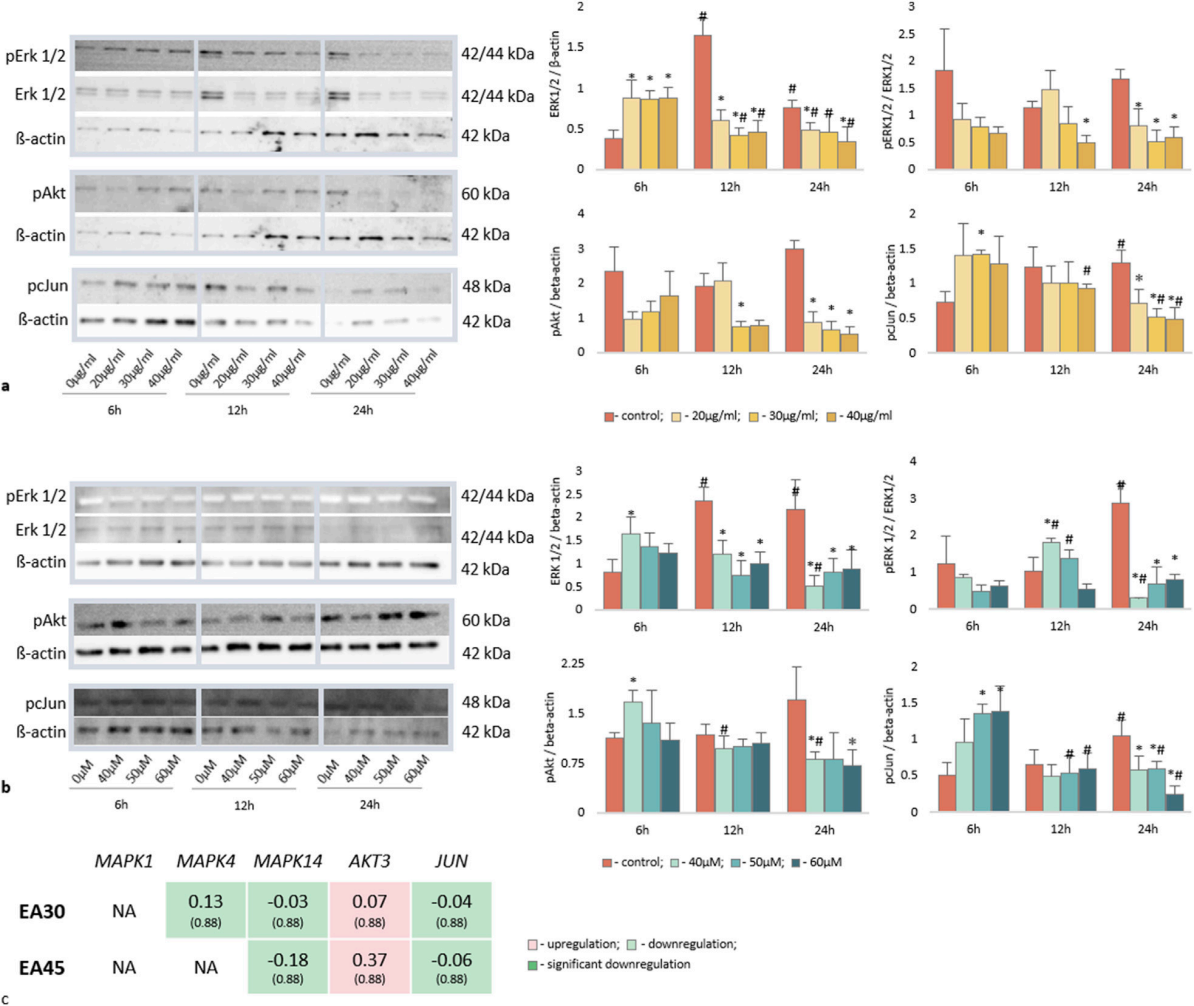
Western blot for key proteins of the MAP Kinase and mTOR pathways expressed in U-87 cells treated with **(a)** EprACN (0, 20, 30, 40 μg/mL) and **(b)** EA (0, 40, 50, 60 µM) over a time period of 6, 12, and 24 h. **(c)** Gene expression of MAPK and PI3K pathways at 24 h ^#^p-value <0.05: time-dependent comparison to control, *p-value <0.05: concentration-dependent protein expression over time. Experiments were performed with 3 replicates.

Western blot analyses revealed that EA, along with EprACN, also modulates key signaling pathways. Figures 4a,b depict the modulation of Erk1/2, c-Jun, and Akt by EprACN and EA over 6, 12, and 24 h. EprACN and EA initially stimulated ERK1/2 formation at 6 h, followed by a downregulation after 12 h and 24 h compared to the control. Higher concentrations of EprACN and EA decreased the ERK phosphorylation at all times. They also downregulated c-Jun phosphorylation after 24 h, with a transient increase observed at 6 h. EprACN downregulated Akt phosphorylation after 6 h, whereas EA insignificantly upregulated it. Significant downregulation was observed after 12 h with EprACN (30 μg/mL), with no further change at 24 h, and with EA (13.29 μg/mL = 40 µM) after 24 h. The RNAseq data of the MAPK and PI3K pathways members’ expression, shown in Figure 4c, demonstrated no significant effect on their transcription.

### 3.4 Multitarget mechanisms of EA-TMZ synergy

**FIGURE 5:**
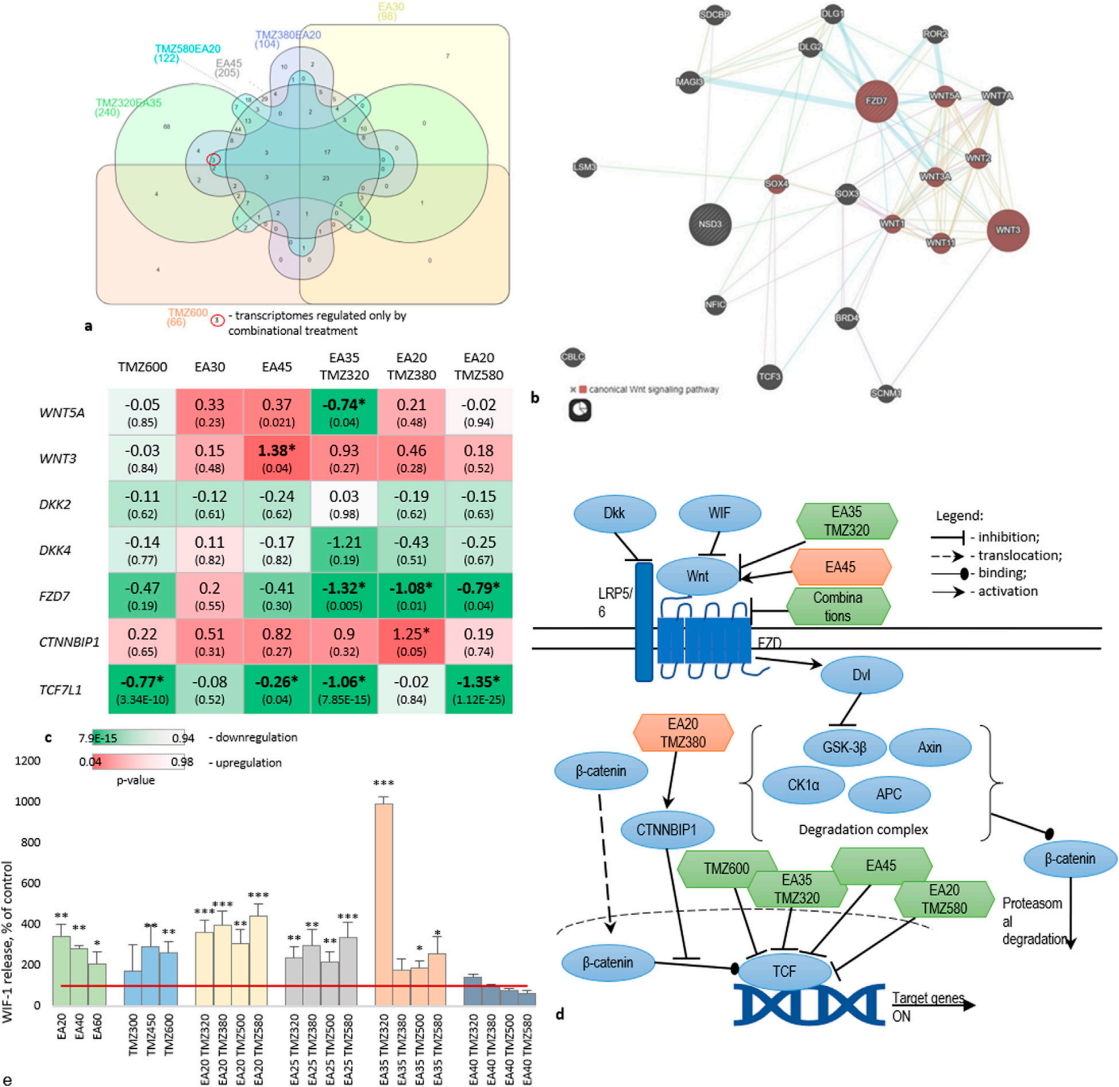
Regulation of Wnt signaling during treatment of U-87 cells with TMZ, EA, and their combinations. **(a)** Venn-diagram (https://www.interactivenn.net/) on significantly regulated genes obtained from the transcriptome analyses. **(b)** Network depicting a possible pathway uniquely regulated by the combinations, obtained from GeneMania (https://genemania.org/). **(c)** Gene expression of Wnt signaling members. Data are presented as logFC. Cell colors represent p-values. **(d)** Schematic representation of the proposed pathway regulated by combinations. **(e)** Level of WIF1 protein release into the culture medium. *p < 0.05, **p < 0.01, ***: p < 0.001. The red line indicates the control. Experiments were performed with 3 replicates.

Expanding the RNA Seq analyses, we compared significantly regulated genes from the GE-analyses for all treatments using Venn diagrams (Figure 5a) to identify unique genes for the combinations. A network built using these genes revealed the canonical Wnt pathway as a pathway regulated by these genes (Figure 5b). The canonical Wnt signaling pathway significantly contributes to the development of resistance to chemo- and radiotherapy in GBM (Zhong and Virshup, 2020). Figure 5c illustrates the expressed genes involved in Wnt signaling upon treatment.

Particularly, EA35TMZ320 significantly downregulated the upstream member *WNT5A,* whereas other treatments did not show a significant effect. Conversely, *WNT3*, a ligand of LRP5/6 (Low-density lipoprotein receptor-related protein 5/6), exhibited significant undesirable upregulation by EA45, whereas *DKK2* and *DKK4*, which are inhibitors of LRP5/6, remained unaffected by any treatment.

All combinations significantly downregulated *FZD7* transcription, which encodes a transmembrane receptor crucial for Wnt downstream. Activation of this receptor by Wnts inhibits the β-catenin degradation complex. Additionally, *CTNNBIP1*, an intracellular member known to prevent β-catenin activity, showed significant upregulation of gene expression by EA20TMZ380.

*TCF7L1*, an intranuclear member of the pathway involved in cell cycle regulation and proliferation, was significantly downregulated by TMZ600, EA45, EA35TMZ320, and EA20TMZ580.

Figure 5d summarizes the interaction of affected targets within the Wnt pathway. The combinations regulated the transcription of more targets compared to single metabolites.

After analyzing the GE of members of the Wnt-signaling pathway under treatment, we wanted to know whether Wnt-signaling is affected on the protein level. Therefore, we measured the Wnt inhibitory factor 1 (WIF1). WIF1 binds to Wnt-proteins, thereby inhibiting the activation of the Wnt-signaling (see also Supplementary Material S7).

Figure 5e shows the release of WIF1 protein by U-87 cells treated with TMZ and EA alone or in combination. Both single metabolites and combinations significantly increased the WIF1 release compared to the control. Combinations demonstrated a dose-dependent increase in WIF1 release, with the highest activity for EA35TMZ320. The effect of TMZ and EA on the WIF1 release showed a concentration-dependent inverse pattern: higher TMZ concentration increases the effect, while higher EA concentration reduces it. Significant up-regulations were observed by the following combinations: EA20TMZ500, EA20TMZ580, EA25TMZ580, EA35TMZ320, EA35TMZ380, and EA30TMZ500. However, EA35TMZ320 showed the highest influence on the WIF1 release.

**FIGURE 6:**
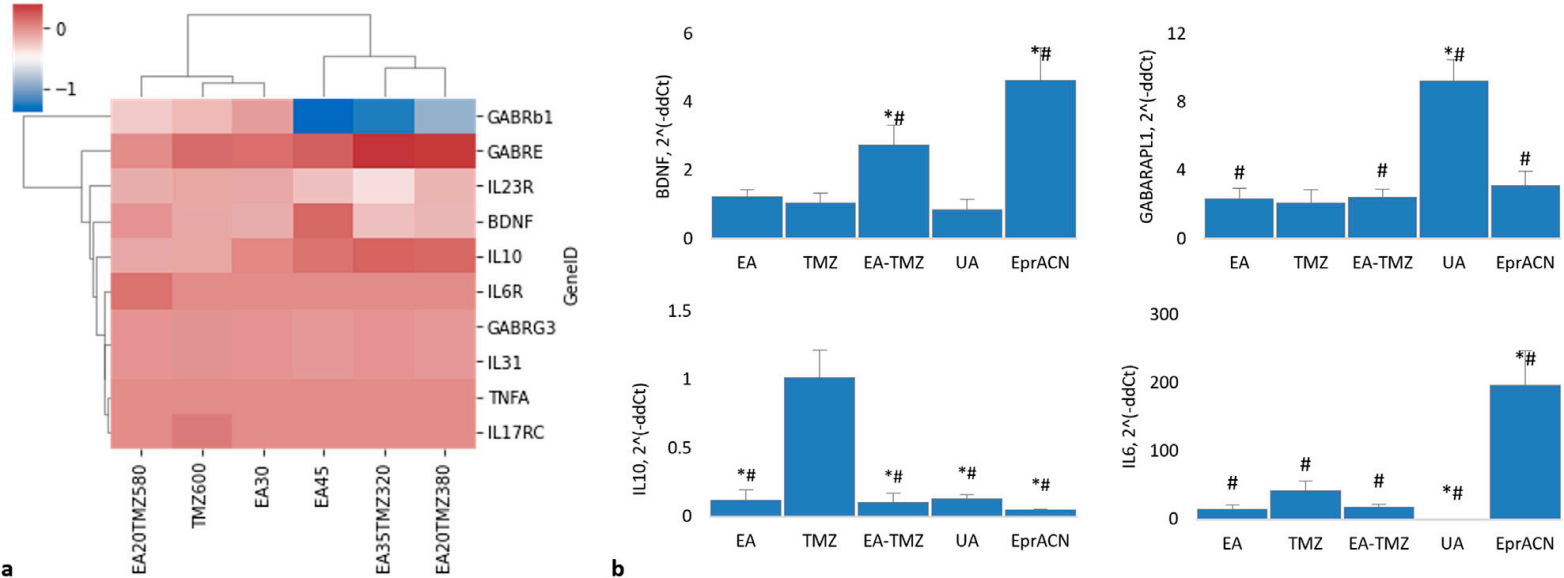
Expression of genes encoding brain-related proteins and cytokines. **(a)** Clustered HeatMap of RNAseq data showing the expression levels of various genes. None of the genes were found to be significantly regulated (p > 0.05). Genes include GABRB1: gamma-aminobutyric acid receptor (GABR) subunit beta 1; GABRE: GABR subunit epsilon; GABRG3: GABR subunit gamma 3; BDNF: brain-derived neurotrophic factor; IL10: interleukin 10; IL6R: interleukin 6 receptor; TNFa: tumor necrosis factor a; IL23R: interleukin 23 receptor; IL31: interleukin 31; IL17RC: interleukin 17 receptor. **(b)** Histograms showing mRNA levels based on qRT-PCR data. Concentrations: EA – 45 μM, TMZ – 600 μM, EA-TMZ – 35–320 μM, UA – 200 μM, and EprACN – 30 μg/mL. GABARAPL1: GABAA receptor-associated protein-like 1. *p < 0.05 comparing to TMZ, #p < 0.05 comparing to untreated control. Experiments were performed with 3 replicates.

The Wnt pathway is a regulatory system interacting directly or indirectly with other signaling pathways, including the NF-kB pathway, as a central pathway in inflammation (Guo et al., 2024). This pathway is essential for connecting inflammation and cancer, as well as for tumor growth and resistance (Guo et al., 2024). For BC, it was shown that NF-kB signaling boosts the growth potential of BC cells and facilitates the spread of tumors (Guo et al., 2024). Therefore, we were interested in the influence of our metabolites and their combinations with TMZ on inflammation. At the same time, we were interested in EA’s reported neuroprotective and anti-inflammatory properties (Lee et al., 2021). Figure 6 shows the influence of the single metabolites and the combinations on the GE of *GABRs*, *BDNF,* and major cytokines regulating inflammatory processes (*IL6, IL10,* and *TNFA*) based on RNA deep sequencing (Figure 6a) and RT-PCR (Figure 6b).

The pro-inflammatory cytokine *TNFA* was neither detected by RNA deep sequencing nor by qRT-PCR. The anti-inflammatory *IL10,* detected by deep sequencing, was not significantly influenced. The RNAseq data showed no regulation of *IL6*. However, qRT-PCR data showed a significantly amplified expression of *IL6* for the single treatment with EprACN or with TMZ, whereas UA downregulated the GE.

RNAseq analysis showed no significant differences in the expression of *GABA* receptor subunits. However, *GABRB1* was downregulated by EA45 (p = 0.07), while *GABARAPL1* was desirably upregulated for all treatments, with the highest expression seen with UA.

RNAseq analysis further revealed insignificant influences of all treatments on *BDNF* transcription. However, in qRT-PCR investigations, *BDNF* was upregulated by EA-TMZ and EprACN and downregulated by UA.

*IL10* gene expression was significantly downregulated by all treatments except TMZ, while *IL6* gene expression was significantly increased by EprACN and decreased by UA.

## 4 Discussion

The effectiveness of standard chemotherapeutics such as temozolomide (TMZ) in glioblastoma (GBM) and paclitaxel (PTX) in breast cancer (BC) is often limited by developing resistance and eventual treatment failure (Das et al., 2021; Lee, 2016). Therefore, we investigated the adjuvant potential of characterized extracts of *E. prunastri* and *C. arbuscula* and their metabolites evernic acid (EA) and usnic acid (UA) alone or in combination with TMZ and PTX for their immunomodulatory and chemosensitivity-increasing potential. HSKF served to evaluate general cytotoxicity and selectivity, even though these cells do not fully replicate the physiological environment or cellular characteristics of normal brain or mammary tissue. U-87 and MCF7 cells have their limitation, too. U-87 cells differ genetically from primary GBM tumors and do not fully recapitulate the intratumoral heterogeneity, stem cell populations, or invasive behavior observed in patient-derived GBM (Xie et al., 2015; Allen et al., 2016). Likewise, MCF7 cells, while representative of HR+ BC, do not model the full spectrum of BC subtypes, particularly TNBC or HER2+ disease, and lack the tumor microenvironment components that influence drug response *in vivo* (Neve et al., 2006; Holliday and Speirs, 2011). Both models are grown in two-dimensional monolayers, which do not capture the complexity of tumor-stroma interactions, hypoxia, or immune modulation present in human tumors. Despite these limitations, the use of U-87 and MCF7 in this study provides a well-established platform for assessing the antiproliferative and resistance-modulating effects of lichen-derived metabolites with findings that can inform subsequent validation in more complex models such as patient-derived cells, organoids, or *in vivo* systems.

The initial experiments revealed a reduction in cellular metabolic activity in U-87 and MCF7 cells after treated with the lichen extracts and their metabolites. However, they were less effective against MCF7 cells compared to PTX. Therefore, experiments with MCF7 cells were discontinued, even though UA had exhibited activity against MCF7 cells in earlier studies (Bačkorová et al., 2011; Hawrył et al., 2020).

For extracts of *E. prunastri,* IC_50_ values of 90.1 μg/mL and 81.8 μg/mL were reported (Bézivin et al., 2004), which is in the range of our results. Effects of EA against MCF7 and U-87 have not been reported earlier. However, previous reports suggest its activity against A-172 and T98G glioblastoma cell lines (Studzińska-Sroka et al., 2021), supporting our findings.

The extracts of *C. arbuscula* were not further investigated due to their non-selective effect. Further experiments focused on the TMZ-resistant U-87 cells (IC_50_ > 500 µM) (Lee, 2016) and the combination of TMZ and EA, since this combination yielded the most promising reduction in cellular metabolic activity, while showing minimal effect on HSKF.

To substantiate the antiproliferative effects, we performed Western blot (WB) analyses of members of the central MAPK families. They play a central role by regulating the cell cycle engine and other proliferation-related proteins (Bézivin et al., 2004; Studzińska-Sroka et al., 2021; Zhang and Liu, 2002). In addition, we measured AKT. In the context of cancer, Akt signaling promotes tumor cell survival, proliferation, growth, and metabolism by activating its downstream effectors. Both the PI3K/Akt and the MEK/ERK pathways cooperate in tumor growth and are involved in the development of therapeutic resistance in GBM cells (Singh et al., 2023).

WB analyses revealed that EprACN and EA reduced the expression and phosphorylation of ERK1/2 over time, explaining the observed decrease in the metabolic activity in U-87 cells and revealing an antiproliferative activity, as in most cells, sustained ERK activation is required to induce cell cycle entry. In Glioma, an aberrant activity of the RAS/MAPK/ERK pathway appears to play a crucial role in the development of gliomas (Stachyra and Grzybowska-Szatkowska, 2025). Even a large proportion of resistance mechanisms are associated with reactivation of the RTK/Raf/Ras/MEK/ERK pathway. Co-treatment with inhibitors targeting these pathways is meanwhile regarded as a compelling strategy to overcome resistance mechanisms in GBM (Yakubov et al., 2025).

Another member of the MAPK-pathway, the so-called proto-oncogene c-Jun, is also central to cancer-altered signalling: an upregulated c-Jun was described for variable tumor cells, specifically in brain tumors, contributing to its malignancy (Blau et al., 2012). Blau et al. demonstrated that the accumulation of c-Jun in tumors is regulated more translationally than transcriptionally (Blau et al., 2012) corresponding to our data with the regulation of c-Jun at the protein but not at the mRNA level.

EprACN and EA downregulated pAkt in a time- and dose-dependent manner corresponding to the reduction in metabolic activity in U-87 cells. This is particularly relevant, given that overexpression and high phosphorylation of Akt correlate with a poor prognosis in glioblastoma patients (Shahcheraghi et al., 2020). Increased pAkt activates transcription factors by phosphorylating GSK3β, leading to its inactivation and subsequent translocation of β-catenin into the nucleus. Both pathways, when activated independently, may contribute to resistance (Manoranjan et al., 2020). Conversely, β-catenin also induces the expression of Akt1 and Akt2 and the phosphorylation of Akt (Zhong and Virshup, 2020). Transcription of *WNT3* was contradictorily upregulated by EA, indicating a limitation as a single treatment at higher concentrations. Nevertheless, the downregulation of Akt by EprACN and EA may contribute to the enhanced sensitivity of U-87 cells to TMZ.

In our combinatory study, GE-profiling of U-87 cells revealed the modulation of multiple components within the Wnt/β-catenin pathway. Combination EA35TMZ320 reduced the transcription of WNT5A, an upstream intracellular member (Yu et al., 2007; Chen et al., 2021) known to play a pro-tumor role in glioma (Zuccarini et al., 2018; Chen et al., 2021) to induce the migration of GBM cells (Lee, 2016) to increase cell proliferation (Yu et al., 2007; Chen et al., 2021) and to correlate with higher WHO histological glioma classification grades (Alkailani et al., 2022). A Wnt5a knockdown inhibited the activity of the GSK3β/β-catenin pathway related to glioma-derived endothelial cell angiogenesis (Chen et al., 2021).

Frizzleds (FZDs) are transmembrane receptors (Alkailani et al., 2022) inhibiting the β-catenin degradation complex (Shahcheraghi et al., 2020). In glioma, FZD7 is upregulated, correlating with poor patient outcomes (Zuccarini et al., 2018). The significant downregulation of *FZD7* GE by all combinations is promising. Alike EA20TMZ380 significantly upregulated the GE of *CTNNBIP1*, which prevents the interaction of β-catenin and TCF (transcription factors) family members. Negative regulation of CTNNBIP1 correlates with higher grades of glioma (Tong et al., 2015).

To support the transcriptional findings, we measured the release of Wnt inhibitory factor (WIF1) protein via ELISA. WIF1 binds to Wnt-proteins, thereby inhibiting Wnt pathway signalling. Both single metabolites and combinations increased the release of WIF1 protein. In contrast, the combination of high concentrations of EA (40 µM) and TMZ (≥380 µM) reduced WIF1 releases, supporting the dose-dependent effects observed in the GE analyses, where higher concentrations induced an upregulation of *WNT3*. This inverse dose-dependent WIF1 regulation underscores the importance of optimized dosing to balance pathway modulation.

Cross-talks between signaling pathways are known to play a role in resistance development f. e. Wnt/ß-catenin signaling activates NF-kB in the cytoplasm, whereas Dvl inhibits NF-kB signaling in the nucleus (Guo et al., 2024). Even more in breast cancer, NF-kB has been confirmed to be a crucial link between resistance signaling pathways (Zhao et al., 2021). Activated NF-kB promotes the production of Wnt, ß-catenin, and ß-TrCP, which can lead to cytokine storms up to death (Guo et al., 2024; Jang et al., 2021). We did not measure NF-kB, but the gene expression of *IL6*, a product of NF-kB, which can activate, via STAT3, cell survival, proliferation, and inflammation (Guo et al., 2024). The resolution of inflammation is regarded as a novel host-focused option to complement existing therapies for glioma (Bazan et al., 2021). IL6 is frequently upregulated in GBM, where it activates JAK/STAT3 signaling to promote tumor cell survival, proliferation, and therapy resistance, and contributes to an immunosuppressive milieu (West et al., 2018). IL10 primarily exerts immunosuppressive effects in the GBM microenvironment by inhibiting effective anti-tumor immune responses and, in some contexts, directly enhancing glioma proliferation via JAK–STAT3 activation (Widodo et al., 2021). EprACN and TMZ increased the transcription of *IL6* (RT-PCR), and *IL10* transcripts were detectable. However, we previously observed that plant ingredients and acetylsalicylic acid stimulated the GE of *IL6* and *IL10* under non-stress conditions, which turned into an anti-inflammatory response under inflammatory conditions. Such cytokine regulations may keep the immune-regulatory system active and influence the cytokine release dynamics (Ulrich-Merzenich et al., 2017). This hypothesis aligns with Ahmad et al.‘s proposition that the simultaneous expression of IL6 and IL10 in tumor tissues improves the survival of breast cancer patients, although underlying mechanisms remain unclear (Ahmad et al., 2018).

We did not observe changes in the GE of *IL4* and *IL8*. However, both exert their most critical actions via crosstalk with immune and endothelial cells. In a tumor-cell-only model, the roles of IL4 and IL8 would likely appear less central compared to their significance in the complex *in vivo* GBM microenvironment (Brat et al., 2005; Losur et al., 2024).

Furthermore, the IL-17B/ IL-17RB pathway has been implicated in tumorigenesis and resistance to anticancer therapies (Briukhovetska et al., 2021). The IL-17A/F, binding to the IL17RC receptor, demonstrated pro-tumoral effects (Briukhovetska et al., 2021). Although its precise role in tumor resistance remains unclear. Our RNAseq data revealed exclusive expression of RNA encoding *IL17RC* in resistant U-87 cells treated with high TMZ concentrations, suggesting that the IL-17 pathway may contribute to the development of resistance in U-87 cells, a finding warranting further study.

Brain-derived neurotrophic factor (BDNF), an endogenous signaling molecule, is involved in the carcinogenesis of glioma (Zheng and Chen, 2020), especially in tumor growth and metastasis in neuroblastoma (Chen et al., 2016), whereas a precursor of BDNF (proBDNF) plays a role in the modulation of cell apoptosis (Xiong et al., 2013). In our study, EA35TMZ320 and EprACN30 induced significant over-expression of *BDNF* without activating GE of the PI3K and MAPK pathway members. We hypothesize that such an effect does not induce cell growth or counteract the reduction in metabolic activity seen in U-87 cells upon different treatments.

GABAA forms a heterotetrameric complex (Huang et al., 2022). The expression of subunits α, β, and γ-subfamilies correlates with the malignancy grade of gliomas (Smits et al., 2012). Patients with high GABAA receptor-associated protein (GABARAPL1) expression levels were reported to have a lower risk for metastasis (Le Grand et al., 2011). In MCF7 cells, GABARAPL was demonstrated to inhibit Dvl2 (disheveled segment polarity protein 2), an inhibitor of the β-catenin degradation complex (Boyer-Guittaut et al., 2014). While TMZ, EA, and TMZ-EA did not affect the GE of Dvl2, TMZ and EA upregulated the GE of *GABARAPL1*. Thus, we suggest that the downregulation of the Wnt signaling cascade observed in our study is likely a direct effect on Wnt signaling members rather than through GABARAPL1.

## 5 Summary and conclusion

This study demonstrates the potential of lichen-derived metabolites, particularly evernic acid (EA), to modulate key pathways associated with TMZ resistance in U-87 cells, suggesting a promising multitarget mechanism for future investigation. EA reduced the metabolic activity of TMZ-resistant U-87 cells, synergizing with TMZ to reduce viability by 75% at optimized ratios. The prediction of ChemGPS that EA acts on tubulin activity was supported by deep sequencing. Mechanistically, EA suppressed GE of oncogenic Wnt/β-catenin signaling while upregulating the protein expression of WIF1 as a central inhibitor of Wnt-signaling. Combinatorial EA-TMZ treatment further modulated MAPK/PI3K pathways, inhibiting ERK1/2, c-Jun, and Akt phosphorylation, which are critical for glioblastoma survival and resistance.

The discovery of IL17RC overexpression in resistant cells underscores a novel pathway implicated in TMZ resistance, warranting further exploration.

Even though present studies lack an *in vivo* validation, findings form a base for subsequent validation in more complex models. Future studies should clarify EA’s direct role in tubulin dynamics, its influence on the IL-17 pathway, on established mechanisms of drug resistance, such as MGMT promoter methylation status, DNA repair pathways, or efflux transporter activity in primary patient-derived cells, as well as *in vivo*, for example, in genetically engineered glioma models (GEGMs) or orthotopic animal models including pharmacodynamic and pharmacokinetic evaluations to explore clinical translation of EA-TMZ combinations. Integrating computational tools like ChemGPS-NP with multi-omics approaches will accelerate the development of natural product-based therapies to address refractory cancers. This work advances the paradigm of combinatorial, mechanism-driven strategies to disrupt resistance-associated pathways and enhance chemosensitivity in oncology.

## Data Availability

The datasets presented in this study can be found in online repositories. The names of the repository/repositories and accession number(s) can be found in the article/Supplementary Material.

## Abbreviations

TMZ, Temozolomide; PXT, Paclitaxel; GBM, glioblastoma; BC, breast cancer; EprDCM, dichloromethane extract from *E. prunastri*; EprACN–acetonitrile extract from *E. prunastri*; CarHex, hexane extract from *C. arbuscula*; CarACN–acetonitrile extract from *C. arbuscula*; EA, evernic acid; UA, usnic acid; TMZ320EA35, combination of TMZ (320 µM = 62.13 μg/mL) with EA (35 µM = 11.63 μg/mL); TMZ380EA20, TMZ (380 µM = 73.78 μg/mL) with EA (20 µM = 6.65 μg/mL); TMZ580EA20, TMZ (580 µM = 112.61 μg/mL) with EA (20 µM = 6.65 μg/mL).
