# Supplementary material for: Phytochemical combinations of lichen Evernia prunastri (L.) Ach. reduce drug resistance to temozolomide but not to paclitaxel in vitro
Source: Front Pharmacol. 2025 Sep 15;16:1633978. doi: 10.3389/fphar.2025.1633978 (PMC12477381; doi:10.3389/fphar.2025.1633978)
Supplement: Supplementary file 1 [file Supplementaryfile1.docx]

**Supplementary Material**

**S1. Western blot analysis**

Western blot analyses were performed as described earlier (54) with minor modifications. In brief, U-87 cells (6x105) were treated with the EprACN extract or EA at different concentrations (20-40 µg/ml and 40-60 µM) over various time intervals (6-24 h). 0.5% DMSO in cell culture medium was the as a vehicle control. After treatment, cells were lysed with lysis buffer (20 mM Tris-Cl, 50 mM NaCl, 50 mM NaF, 10 mM EDTA, 20 mM Na4P2O7) and the lysates were centrifuged at 12,000 rpm for 10 min. Protein concentrations were measured by Micro BСA Assay Kit according to the manufacturer’s recommendations. Proteins (40 µg) were separated on 10% SDS-PAGE and transferred to the nitrocellulose membrane by Trans-Blot® Turbo™ (Bio-Rad, USA). The membranes were blocked with 1% BSA (bovine serum albumin) in TBST (Tris-buffered saline with 0.05% Tween20). Further membranes were incubated with the primary antibody (Ab) overnight, followed by washing and incubation with the secondary Ab for 1h. The primary Ab was detected using the respective Ab and visualized by Western lightning chemiluminescence (ECL, Perkin Elmer Life Science) after extensive washing of the membranes. Bands were evaluated by a Peqlab Gel-documentation station and quantified with the ImageJ software (NIH). The amount of proteins was calculated in relation to β-actin in the sample, and the amount of phosphorylated proteins was calculated in relation to the unphosphorylated proteins.

**Table S1. List of antibodies for Western Blot**

| Name | Symbol | ID | Concentration | Company |
| --- | --- | --- | --- | --- |
| phospho-AKT rabbit polyclonal antibody | pAkt | sc-293125 | 1:2000 | Santa Cruz Biotechnology, USA |
| phospho-p44/42 (THR202/Tyr204) rabbit polyclonal antibody | pErk 1/2 | sc-81492 | 1:2000 | Santa Cruz Biotechnology, USA |
| ERK 1/2 mouse clonal antibody | Erk 1/2 | sc-514302 | 1:2000 | Santa Cruz Biotechnology, USA |
| phospho-c-Jun (Ser73) rabbit polyclonal antibody | p-cJun | 9164 | 1:2000 | Cell Signaling Technology, USA |
| β-Actin rabbit polyclonal antibody | β-Actin | sc-30656 | 1:2000 | Santa Cruz Biotechnology, USA |
| goat anti-rabbit IgG-HRP conjugated to horse radish peroxidase  goat anti-mouse IgG-HRP conjugated to horse radish peroxidase | Monoclonal Secondary Antibodies.  Monoclonal Secondary Antibodies | sc-2004  sc-2005 | 1:2000  1:2000 | Santa Cruz Biotechnology, USA  Santa Cruz Biotechnology, USA |

**S2. Design of the combination treatment**

**Table S2. Design of the cell stimulation with different combinations:** a) TMZ+EP; b) TMZ+EA/UA; c) PTX+EP; d) PTX+EA/UA

| # | EP (ug/ml) | TMZ (uM) |  | # | EA/UA (uM) | TMZ (uM) |  | # | EP (ug/ml) | PTX (uM) |  | # | EA/UA (uM) | PTX (uM) |
| --- | --- | --- | --- | --- | --- | --- | --- | --- | --- | --- | --- | --- | --- | --- |
| C1 | 6.25 | 50 |  | C1 | 12.5 | 50 |  | C1 | 6.25 | 6.25 |  | C1 | 12.5 | 6.25 |
| C2 | 12.5 | 50 |  | C2 | 25 | 50 |  | C2 | 12.5 | 6.25 |  | C2 | 25 | 6.25 |
| C3 | 25 | 50 |  | C3 | 50 | 50 |  | C3 | 25 | 6.25 |  | C3 | 50 | 6.25 |
| C4 | 50 | 50 |  | C4 | 100 | 50 |  | C4 | 50 | 6.25 |  | C4 | 100 | 6.25 |
| C5 | 100 | 50 |  | C5 | 200 | 50 |  | C5 | 100 | 6.25 |  | C5 | 200 | 6.25 |
| C6 | 6.25 | 100 |  | C6 | 12.5 | 100 |  | C6 | 6.25 | 12.5 |  | C6 | 12.5 | 12.5 |
| C7 | 12.5 | 100 |  | C7 | 25 | 100 |  | C7 | 12.5 | 12.5 |  | C7 | 25 | 12.5 |
| C8 | 25 | 100 |  | C8 | 50 | 100 |  | C8 | 25 | 12.5 |  | C8 | 50 | 12.5 |
| C9 | 50 | 100 |  | C9 | 100 | 100 |  | C9 | 50 | 12.5 |  | C9 | 100 | 12.5 |
| C10 | 100 | 100 |  | C10 | 200 | 100 |  | C10 | 100 | 12.5 |  | C10 | 200 | 12.5 |
| C11 | 6.25 | 200 |  | C11 | 12.5 | 200 |  | C11 | 6.25 | 25 |  | C11 | 12.5 | 25 |
| C12 | 12.5 | 200 |  | C12 | 25 | 200 |  | C12 | 12.5 | 25 |  | C12 | 25 | 25 |
| C13 | 25 | 200 |  | C13 | 50 | 200 |  | C13 | 25 | 25 |  | C13 | 50 | 25 |
| C14 | 50 | 200 |  | C14 | 100 | 200 |  | C14 | 50 | 25 |  | C14 | 100 | 25 |
| C15 | 100 | 200 |  | C15 | 200 | 200 |  | C15 | 100 | 25 |  | C15 | 200 | 25 |
| C16 | 6.25 | 400 |  | C16 | 12.5 | 400 |  | C16 | 6.25 | 50 |  | C16 | 12.5 | 50 |
| C17 | 12.5 | 400 |  | C17 | 25 | 400 |  | C17 | 12.5 | 50 |  | C17 | 25 | 50 |
| C18 | 25 | 400 |  | C18 | 50 | 400 |  | C18 | 25 | 50 |  | C18 | 50 | 50 |
| C19 | 50 | 400 |  | C19 | 100 | 400 |  | C19 | 50 | 50 |  | C19 | 100 | 50 |
| C20 | 100 | 400 |  | C20 | 200 | 400 |  | C20 | 100 | 50 |  | C20 | 200 | 50 |
| C21 | 6.25 | 800 |  | C21 | 12.5 | 800 |  | C21 | 6.25 | 100 |  | C21 | 12.5 | 100 |
| C22 | 12.5 | 800 |  | C22 | 25 | 800 |  | C22 | 12.5 | 100 |  | C22 | 25 | 100 |
| C23 | 25 | 800 |  | C23 | 50 | 800 |  | C23 | 25 | 100 |  | C23 | 50 | 100 |
| C24 | 50 | 800 |  | C24 | 100 | 800 |  | C24 | 50 | 100 |  | C24 | 100 | 100 |
| C25 | 100 | 800 |  | C25 | 200 | 800 |  | C25 | 100 | 100 |  | C25 | 200 | 100 |

**S3. Quantitative RT-PCR (qRT-PCR)**

RNA samples analyzed by RNASeq were used for the quantitative reverse transcription (qRT) – PCR (qRT-PCR). cDNA was performed with 500 ng of total RNA using the cDNA Reverse Transcription Kit (Applied Biosystem) following the manufacturer’s recommendations. The qRT-PCR was carried out using predesigned TaqMan® primers (FAM™ dye-labeled; Life Technologies GmbH, Darmstadt, Germany). The TaqMan® gene expression arrays were performed in 96-well plates (3 replicates each) on a TaqMan® Thermal Cycler 7300 System with the TaqMan gene expression master mix. qRT-PCR conditions were as follows: 50°: 2 min (1x); 95°:10 min (1x); 95°:15 sec (40x); 60°: 1 min (40x). The gene-specific primers GABARAPL1, BDNF, IL-6, IL-10, and 18sRNA (see table below) were used for the amplification. The expression of each gene was calculated as 2-ΔΔCt as described earlier (58).

**Table S3. List of Genes and TAQ-Man Primers**

| Gene Name | Gene Symbol | Gene ID | TAQ-Man Primer ID |
| --- | --- | --- | --- |
| GABA type A receptor-associated protein-like 1 | GABARAPL1 | 23710 | Hs05557280_s1 |
| Brain-derived neurotrophic factor | BDNF | 627 | Hs02718934_s1 |
| Interleukin 6 | IL-6 | 3569 | Hs00985639_m1 |
| Interleukin 10 | IL-10 | 3586 | Hs00961622_m2 |
| Eukaryotic 18S rRNA  Endogenous Control (FAM™/MGB) | RNA18S5 | - | Hs03928990_g1 |

**S4. Initial screening of extract and compound concentrations by resazurin assay**

**
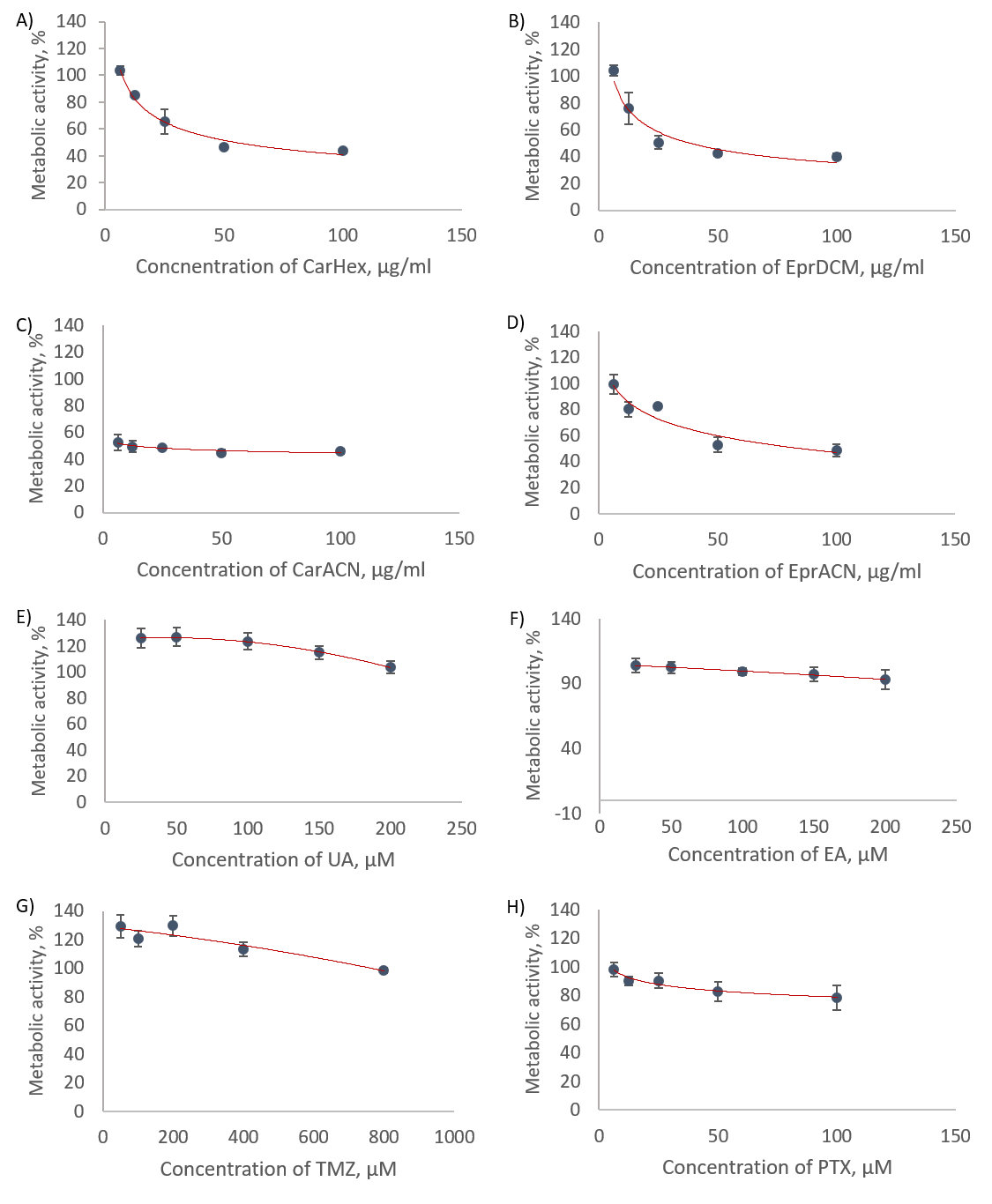
**

**Figure S4.1. Effect of extracts on metabolic activity of HSKF cells:** a) *C. arbuscula* Hexane extract (IC_50_=44 µg/ml); b) *C. arbuscula* acetonitrile extract (IC_50_=16 µg/ml); c) *E. prunastri* dichloromethane extract (IC_50_=29 µg/ml); d) *E. prunastri* acetonitrile extract (IC_50_=79 µg/ml); e) usnic acid (IC_50_>200 µM); f) evernic acid (IC_50_>200 µM); temozolomide (IC_50_>800 µM); paclitaxel (IC_50_>100 µM).


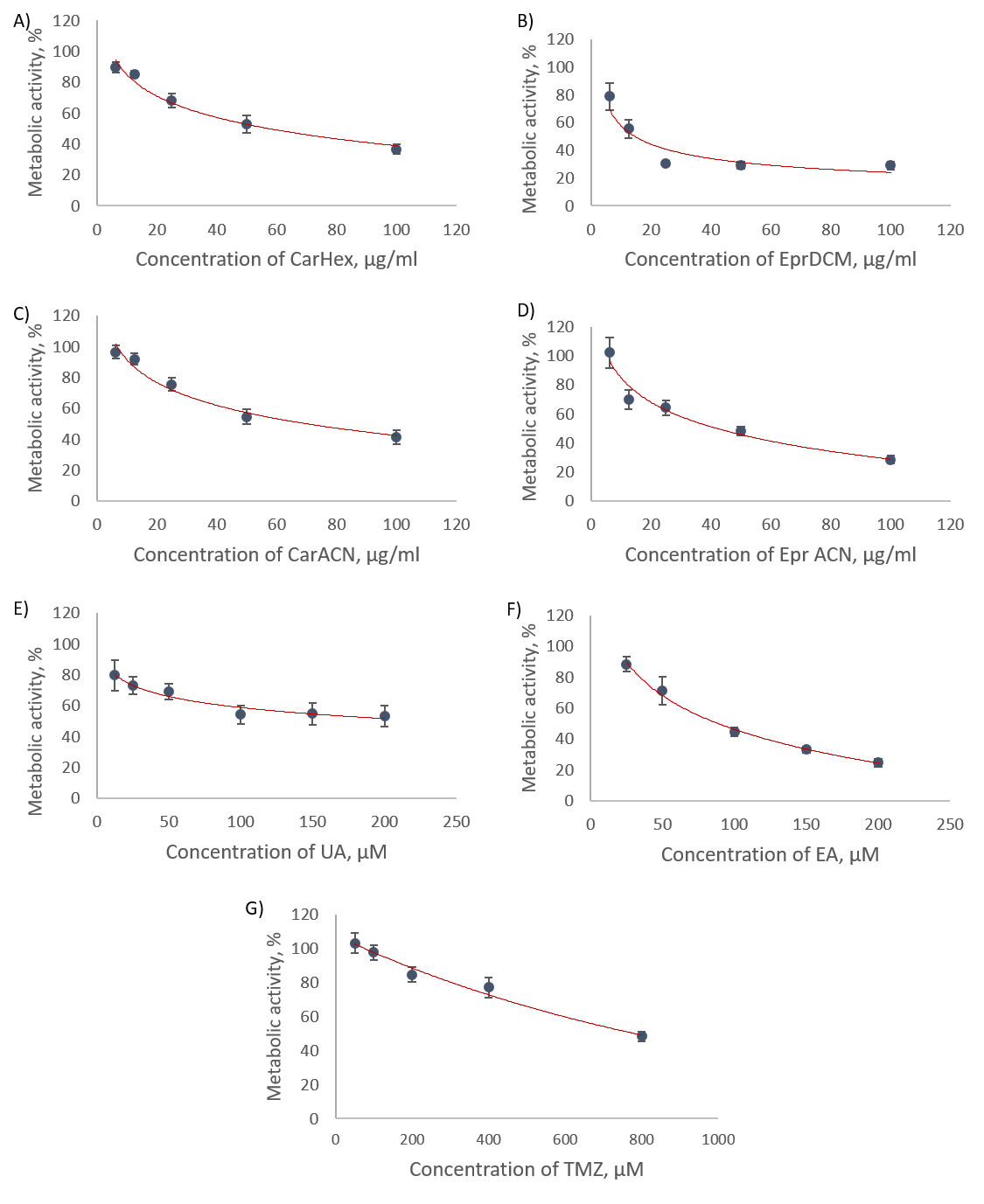


**Figure S4.2. Effect of extracts on metabolic activity of U-87 cells:** a) *C. arbuscula* Hexane extract (IC50=35 µg/ml); b) *C. arbuscula* acetonitrile extract (IC50=33 µg/ml); c) *E. prunastri* dichloromethane extract (IC50=13 µg/ml); d) *E. prunastri* acetonitrile extract (IC50=31 µg/ml); e) evernic acid (IC50=62 µM); f) usnic acid (IC50=203 µM); g) temozolomide (IC50=593 µM).


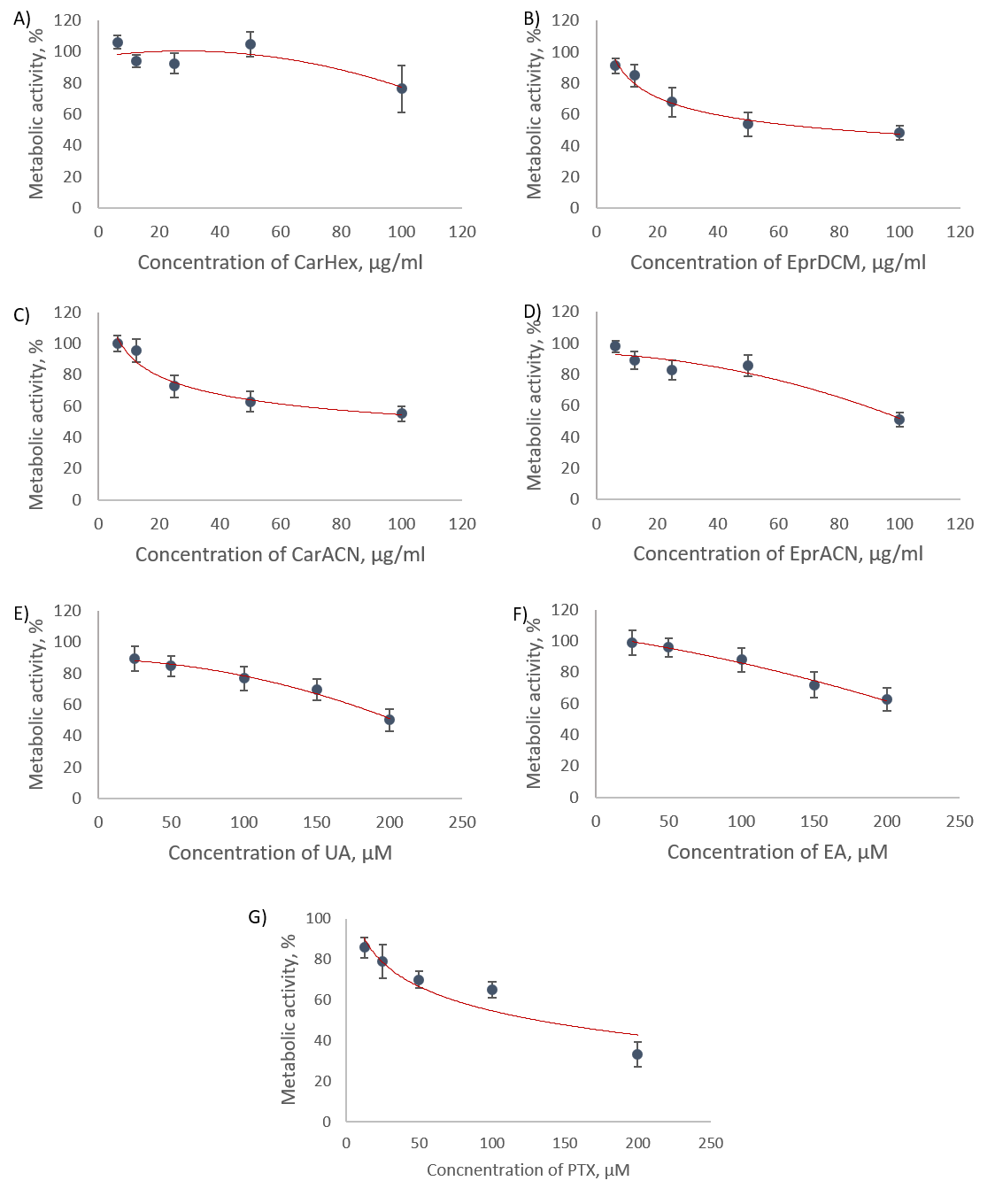


**Figure S4.3. Effect of extracts on metabolic activity of MCF7 cells:** a) *C. arbuscula* Hexane extract (IC50=85 µg/ml); b) *C. arbuscula* acetonitrile extract (IC50=67 µg/ml); c) *E. prunastri* dichloromethane extract (IC50=72 µg/ml); d) *E. prunastri* acetonitrile extract (IC50=107 µg/ml); e) evernic acid (IC50=260 µM); f) usnic acid (IC50=167 µM); g) paclitaxel (IC50=136 µM).

**S5. Genes encoding modes of action predicted by ChemGPS-NP**

**Table S5. Expression of genes encoding modes of action predicted by ChemGPS-NP**

| Gene ID | TMZ600 | EA30 | EA45 | EA35TMZ320 | EA20TMZ380 | EA20TMZ580 |
| --- | --- | --- | --- | --- | --- | --- |
| TOP1 | NA | NA | NA | NA | NA | NA |
| TOP3B | \| 0.237 \| 0.58 \| \| --- \| --- \|   (0.58) | 0.449  (0.326) | 0.249  (0.744) | 0.026  (0.98) | 0.585  (0.372) | 0.149  (0.785) |
| TUBB6 | **-0.62** | **-0.73** | -0.33 | -0.17 | -0.40 | **-0.75** |
|  | (0.02) | (0.01) | (0.26) | (0.57) | (0.17) | (0.01) |
| TUBB2B | **-1.05** | **-0.99** | -0.56 | -0.67 | -0.22 | **-1.42** |
|  | (0.03) | (0.04) | (0.34) | (0.31) | (0.68) | (0.01) |
| TUBA4B | 0.03 | 0.22 | -0.03 | -0.29 | 0.08 | -0.51 |
|  | (0.95) | (0.65) | (0.97) | (0.76) | (0.90) | (0.39) |
| TBCB | 0.27 | 0.11 | -0.08 | 1.15 | 0.33 | -0.18 |
|  | (0.47) | (0.76) | (0.91) | (0.26) | (0.57) | (0.69) |
| TPPP3 | -0.03 | -0.04 | -0.06 | -0.04 | -0.05 | -0.04 |
|  | (0.84) | (0.80) | (0.88) | (0.95) | (0.87) | (0.87) |

*Data are presented as log2Fold change (p-value).

^‡^ NA – the gene is “not available” or the gene was excluded from analysis because it contained an extreme count outlier – large counts (during the gene expression analysis in Galaxy)

Abbreviations: TUBB6: tubulin beta-6 chain; TUBB2B: tubulin beta-2B chain; TUBA4B: putative tubulin-like protein alpha-4B; TPPP3: tubulin polymerization-promoting protein family member 3; TBCB: tubulin-folding cofactor B.

**S6. Gene ontology analyses**

**Table S6.1 Gene ontology analysis** of the gene expression in U-87 cell line during treatment with EA30

| term name | z-score | combined score | p value | log10P | geneset |
| --- | --- | --- | --- | --- | --- |
| glycolytic process through glucose-6-phosphate (GO:0061620) | 17.60 | 452.20 | 6.9E-12 | 11.16 | downregulated |
| glucose catabolic process to pyruvate (GO:0061718) | 17.60 | 452.20 | 6.9E-12 | 11.16 | downregulated |
| canonical glycolysis (GO:0061621) | 17.60 | 452.20 | 6.9E-12 | 11.16 | downregulated |
| negative regulation of cellular process (GO:0048523) | 2.85 | 53.07 | 8.0E-09 | 8.10 | downregulated |
| negative regulation of transcription, DNA-templated (GO:0045892) | 2.41 | 43.87 | 1.2E-08 | 7.90 | downregulated |
| cytoskeleton-dependent cytokinesis (GO:0061640) | 7.43 | 133.12 | 1.7E-08 | 7.78 | downregulated |
| glycolytic process (GO:0006096) | 13.91 | 233.66 | 5.1E-08 | 7.29 | downregulated |
| ATP generation from ADP (GO:0006757) | 13.33 | 218.81 | 7.5E-08 | 7.13 | downregulated |
| mitotic sister chromatid segregation (GO:0000070) | 6.34 | 101.16 | 1.2E-07 | 6.93 | downregulated |
| pyruvate metabolic process (GO:0006090) | 8.70 | 136.16 | 1.6E-07 | 6.80 | downregulated |
| mitotic cell cycle phase transition (GO:0044772) | 3.80 | 58.95 | 1.8E-07 | 6.74 | downregulated |
| negative regulation of cellular macromolecule biosynthetic process (GO:2000113) | 2.66 | 40.29 | 2.6E-07 | 6.59 | downregulated |
| cellular response to decreased oxygen levels (GO:0036294) | 6.88 | 100.03 | 4.8E-07 | 6.32 | downregulated |
| gluconeogenesis (GO:0006094) | 8.78 | 125.64 | 6.1E-07 | 6.21 | downregulated |
| IRE1-mediated unfolded protein response (GO:0036498) | 12.63 | 432.44 | 1.4E-15 | 14.87 | upregulated |
| protein exit from endoplasmic reticulum (GO:0032527) | 21.05 | 542.03 | 6.6E-12 | 11.18 | upregulated |
| response to endoplasmic reticulum stress (GO:0034976) | 6.79 | 154.31 | 1.4E-10 | 9.87 | upregulated |
| retrograde protein transport, ER to cytosol (GO:0030970) | 20.00 | 453.60 | 1.4E-10 | 9.85 | upregulated |
| endoplasmic reticulum to cytosol transport (GO:1903513) | 20.00 | 405.61 | 1.6E-09 | 8.81 | upregulated |
| ubiquitin-dependent ERAD pathway (GO:0030433) | 8.28 | 148.35 | 1.6E-08 | 7.79 | upregulated |
| ERAD pathway (GO:0036503) | 6.75 | 112.95 | 5.4E-08 | 7.26 | upregulated |
| PERK-mediated unfolded protein response (GO:0036499) | 20.00 | 309.25 | 1.9E-07 | 6.72 | upregulated |
| regulation of primary metabolic process (GO:0080090) | 4.03 | 46.19 | 1.0E-05 | 4.98 | upregulated |
| posttranslational protein targeting to endoplasmic reticulum membrane (GO:0006620) | 22.86 | 257.65 | 1.3E-05 | 4.90 | upregulated |
| proteasome-mediated ubiquitin-dependent protein catabolic process (GO:0043161) | 2.89 | 32.05 | 1.5E-05 | 4.82 | upregulated |
| interleukin-1-mediated signaling pathway (GO:0070498) | 4.63 | 49.06 | 2.5E-05 | 4.60 | upregulated |
| response to organonitrogen compound (GO:0010243) | 6.27 | 64.25 | 3.6E-05 | 4.45 | upregulated |
| tRNA aminoacylation (GO:0043039) | 7.00 | 68.87 | 5.3E-05 | 4.27 | upregulated |
| protein N-linked glycosylation (GO:0006487) | 4.93 | 46.32 | 8.3E-05 | 4.08 | upregulated |
|  |  |  |  |  |  |

**Table S6.2 Gene ontology analysis** of the gene expression in U-87 cell line during treatment with EA45

| term name | z-score | combined score | pvalue | log10P | geneset |
| --- | --- | --- | --- | --- | --- |
| mitotic cell cycle phase transition (GO:0044772) | 4.52 | 98.71 | 3.4E-10 | 9.47 | downregulated |
| regulation of mitotic cell cycle phase transition (GO:1901990) | 4.57 | 85.40 | 7.5E-09 | 8.12 | downregulated |
| mitotic sister chromatid segregation (GO:0000070) | 6.83 | 123.30 | 1.4E-08 | 7.84 | downregulated |
| regulation of cell cycle G2/M phase transition (GO:1902749) | 6.67 | 118.22 | 2.0E-08 | 7.70 | downregulated |
| establishment of mitotic spindle localization (GO:0040001) | 15.56 | 244.77 | 1.5E-07 | 6.83 | downregulated |
| mitotic cytokinesis (GO:0000281) | 7.46 | 114.95 | 2.0E-07 | 6.69 | downregulated |
| extracellular matrix organization (GO:0030198) | 3.67 | 54.69 | 3.3E-07 | 6.48 | downregulated |
| G1/S transition of mitotic cell cycle (GO:0000082) | 5.33 | 79.19 | 3.6E-07 | 6.45 | downregulated |
| establishment of spindle orientation (GO:0051294) | 13.33 | 193.39 | 5.0E-07 | 6.30 | downregulated |
| establishment of mitotic spindle orientation (GO:0000132) | 17.14 | 245.59 | 6.0E-07 | 6.22 | downregulated |
| mitotic chromosome condensation (GO:0007076) | 16.00 | 221.39 | 9.8E-07 | 6.01 | downregulated |
| chromosome condensation (GO:0030261) | 12.17 | 168.02 | 1.0E-06 | 5.99 | downregulated |
| cytoskeleton-dependent cytokinesis (GO:0061640) | 6.29 | 85.59 | 1.2E-06 | 5.91 | downregulated |
| glycolytic process through glucose-6-phosphate (GO:0061620) | 11.20 | 147.53 | 1.9E-06 | 5.72 | downregulated |
| canonical glycolysis (GO:0061621) | 11.20 | 147.53 | 1.9E-06 | 5.72 | downregulated |
| response to endoplasmic reticulum stress (GO:0034976) | 10.19 | 452.34 | 5.2E-20 | 19.28 | upregulated |
| IRE1-mediated unfolded protein response (GO:0036498) | 13.33 | 496.24 | 6.9E-17 | 16.16 | upregulated |
| interleukin-1-mediated signaling pathway (GO:0070498) | 8.42 | 246.74 | 1.9E-13 | 12.72 | upregulated |
| NIK/NF-kappaB signaling (GO:0038061) | 9.00 | 249.51 | 9.1E-13 | 12.04 | upregulated |
| regulation of cellular amine metabolic process (GO:0033238) | 11.76 | 324.44 | 1.1E-12 | 11.98 | upregulated |
| regulation of cellular amino acid metabolic process (GO:0006521) | 11.76 | 324.44 | 1.1E-12 | 11.98 | upregulated |
| proteasome-mediated ubiquitin-dependent protein catabolic process (GO:0043161) | 4.40 | 117.83 | 2.3E-12 | 11.63 | upregulated |
| regulation of cellular ketone metabolic process (GO:0010565) | 9.84 | 243.06 | 1.9E-11 | 10.73 | upregulated |
| cellular response to hypoxia (GO:0071456) | 6.06 | 139.03 | 1.1E-10 | 9.96 | upregulated |
| cellular response to interleukin-1 (GO:0071347) | 5.60 | 125.96 | 1.7E-10 | 9.77 | upregulated |
| regulation of primary metabolic process (GO:0080090) | 5.76 | 126.55 | 2.8E-10 | 9.55 | upregulated |
| negative regulation of G2/M transition of mitotic cell cycle (GO:0010972) | 9.03 | 198.01 | 3.0E-10 | 9.52 | upregulated |
| negative regulation of mitotic cell cycle phase transition (GO:1901991) | 7.59 | 157.88 | 9.4E-10 | 9.03 | upregulated |
| negative regulation of cell cycle G2/M phase transition (GO:1902750) | 8.12 | 165.66 | 1.4E-09 | 8.86 | upregulated |
| ubiquitin-dependent ERAD pathway (GO:0030433) | 8.97 | 182.51 | 1.4E-09 | 8.84 | upregulated |

**S7: Wnt-inhibitory Factor 1 protein release in U-87 cells**

**Table S7.1. Design of combination treatment for investigation of the WIF1 protein expression**

|  | C1 | C2 | C3 | C4 | C5 | C6 | C7 | C8 | C9 | C10 | C11 | C12 | C13 | C14 | C15 | C16 |
| --- | --- | --- | --- | --- | --- | --- | --- | --- | --- | --- | --- | --- | --- | --- | --- | --- |
| TMZ (uM) | 320 | 380 | 500 | 580 | 320 | 380 | 500 | 580 | 320 | 380 | 500 | 580 | 320 | 380 | 500 | 580 |
| EA (uM) | 20 | 20 | 20 | 20 | 25 | 25 | 25 | 25 | 35 | 35 | 35 | 35 | 40 | 40 | 40 | 40 |

.

**Table S7.2. The significance levels of the comparisons within and between the different groups in the WIF-1 release**
